# Ionic liquids meet lipid bilayers: a state-of-the-art review

**DOI:** 10.1007/s12551-023-01173-3

**Published:** 2024-01-02

**Authors:** Antonio Benedetto

**Affiliations:** 1 School of Physics, University College Dublin, Dublin, Ireland; 2 Conway Institute of Biomolecular and Biomedical Research, University College Dublin, Dublin, Ireland; 3 Department of Science, University of Roma Tre, Rome, Italy; 4 Laboratory for Neutron Scattering, Paul Scherrer Institute, Villigen, Switzerland

**Keywords:** Ionic liquids, Lipid bilayers, Membrane biophysics, Ionic liquids biophysics

## Abstract

In the past 25 years, a vast family of complex organic salts known as room-temperature ionic liquids (ILs) has received increasing attention due to their potential applications. ILs are composed by an organic cation and either an organic or inorganic anion, and possess several intriguing properties such as low vapor pressure and being liquid around room temperature. Several biological studies flagged their moderate-to-high (cyto)-toxicity. Toxicity is, however, also a synonym of affinity, and this boosted a series of biophysical and chemical-physical investigations aimed at exploiting ILs in bio-nanomedicine, drug-delivery, pharmacology, and bio-nanotechnology. Several of these investigations focused on the interaction between ILs and lipid membranes, aimed at determining the microscopic mechanisms behind their interaction. This is the focus of this review work. These studies have been carried out on a variety of different lipid bilayer systems ranging from 1-lipid to 5-lipids systems, and also on cell-extracted membranes. They have been carried out at different chemical-physical conditions and by the use of a number of different approaches, including atomic force microscopy, neutron and X-ray scattering, dynamic light scattering, differential scanning calorimetry, surface quartz microbalance, nuclear magnetic resonance, confocal fluorescence microscopy, and molecular dynamics simulations. The aim of this “2023 Michèle Auger Award” review work is to provide the reader with an *up-to-date* overview of this fascinating research field where “ILs meet lipid bilayers (aka biomembranes),” with the aim to boost it further and expand its cross-disciplinary edges towards novel high-impact ideas/applications in pharmacology, drug delivery, biomedicine, and bio-nanotechnology.

## Introduction

**Fig. 1:**
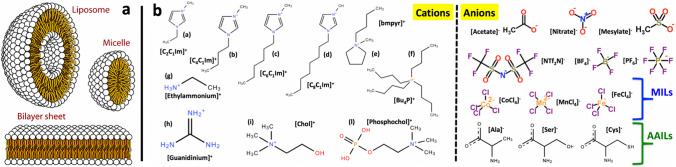
**a** Representative image of the common supramolecular structures of phospholipids in solutions. **b** Cations and anions from selected/common ILs, including magnetic ILs (MILs) and amino acid ILs (AAILs) subcategories. Taken from (Benedetto and Ballone 2018) and reproduced with permission from the publisher

Several key processes for life, such as cell differentiation, migration, adhesion, and apoptosis are regulated by the ability of cells to sense and respond to the mechano-elasticity of the surrounding environment through their membranes (Jaalouk and Lammerding 2009). Sensing and response, in particular, are mediated by changes in the cell membrane fluidity and viscoelasticity, which control the activity of a variety of membrane proteins and the formation of bio-functional (raft) domains (Nomura et al. 2012). Key components of cell membranes are the phospholipids, i.e., amphiphilic molecules composed of a hydrophilic polar head and hydrophobic nonpolar tails (Phillips et al. 2013). In cell membranes, they are organized into a supramolecular bilayer structure in which other biomolecules reside, including a variety of membrane proteins. Cell membranes are made by a variety of different phospholipids that are heterogeneously distributed across the bilayer and directly involved in several major biochemical processes as, for example, cell apoptosis, cell signalling, and cell recognition (Sezgin et al. 2017). Due to their amphiphilic character, phospholipids form a variety of supramolecular structures such as micelles, vesicles, and bilayers (Fig. 1a). Phospholipid vesicles of nanometric size are also referred to as liposomes or as phospholipid nanoparticles (NPs). NPs of this kind have been extensively studied for the last 50 + years as potential carriers for drug delivery (Hou et al. 2021). Due to the similar structure and composition, they are able to fuse with cell membranes and release their content into the cytoplasm. In the last couple of years, this extraordinary research effort has come to fruition: these NPs have been used as drug nano-carriers in the mRNA-based Covid-19 vaccines (Horejs 2021). It is envisaged that phospholipid NPs will be a key actor of the bio-nanotechnology revolution of the current decade. However, major challenges for the further use of this technology include (i) targeting specific cells, for example cancer cells in the body of a patient, and (ii) controlling the release of the drug (Truong et al. 2019). In summary, phospholipid bilayers are key components of cells and key players of the current “bio-nanotechnology revolution.” Hence, controlling properties such as their fluidity and viscoelasticity, is of utmost importance. Room temperature ionic liquids (ILs)—a vast class of organic electrolyte — can serve to this cause and can be the additional handle required for the development of breakthrough applications in bio-nanotechnology based on lipids.

## Ionic liquids

In the last 20 years, a new family of complex organic salts known as ILs have received increasing attention for their potential applications as solvents, non-aqueous electrolytes, high-performance lubricants, and advanced engineering materials (Welton 1999; MacFarlane et al. 2016). ILs are a vast class of organic electrolytes composed by an organic cation and either an organic or inorganic anion (Fig. 1b), which possess several intriguing properties as, for example, having low vapor pressure and being liquid around room temperature (Hallett and Welton 2011). Historically, the first IL has been probably discovered in 1914 by Paul Walden, followed then in 1951 by Hurley and Weir. However, it is only by the end of the twentieth century that ILs were coming to the attention of a wider audience (Welton 2018). In light of the potential (industrial) use of ILs, several biological studies have been performed with the aim to assess the toxicity of these organic ionic species towards cells and organisms. The original low-environmental impact shown by the first ILs was not confirmed as a common characteristic of these new liquid electrolytes that turned out to have moderate-to-high toxicity towards several micro-organisms, plants, cell lines, and potentially humans (Egorova and Ananikov 2014; Egorova et al. 2020). For example, ILs based on amino-acids (Benedetto et al. 2014a), which were supposed to be more bio-friendly, turned out to have higher toxicity level than their common counterparts (Egorova et al. 2015). It has been shown that the toxicity (i) depends mainly on IL-cations with, in general, a minor impact of IL-anions (Stolte et al. 2006), (ii) can be time-dependent (Li et al. 2015), (iii) increases with increasing IL-concentration and IL-chain length (Ranke et al. 2004), and (iv) positively correlates with the degree of IL-cation lipophilicity (Vraneš et al. 2019), suggesting membrane damage as one major potential mechanism of toxicity.

Toxicity is, however, also a synonym of affinity, and this have stimulated, in turn, a series of biological and chemical-physical investigations aimed at using the ability of ILs to interact with biosystems to develop applications in bio-nanomedicine and bio-nanotechnology (Egorova et al. 2017; Benedetto and Ballone 2018; Banerjee et al. 2018; Tanner et al. 2020; Curreri et al. 2021; Kumari et al. 2021a; Pillai et al. 2022a; Hu et al. 2023). For example, it has been shown that the oral delivery of a choline-based IL prevents the absorption of fat molecules through intestinal tissue in rats (Nurunnabi et al. 2019). More in general, there are several studies highlighting the specificity of ILs towards cancer cells and their ability to kill those cells leaving healthy cells almost unaffected (Kumar and Malhotra 2009; Stromyer et al. 2020). In this context, it has been shown that tri-n-butyl-n-hexadecyl-phosphonium bromide is approximately 100-times more toxic against HeLa cells than several ammonium-based ILs, but it remains inactive against human chronic myelogenous leukemia K562 cells; complementarily, triphenyl-alkyl-phosphonium iodides resulted in being inactive against HeLa cells, but very active against K562 cells (Bachowska et al. 2012). Moreover, non-toxic ILs or sub-toxic IL-doses are also showing promising applications. For example, it has been shown that some non-toxic ammonium-based ILs, as well as novel di-cationic double-tail lipid-mimic imidazolium-ILs, can be used as gene delivery vehicles since they could electrostatically interact with negatively charged DNA or RNA molecules (Paulisch et al. 2020). Toxicity of ILs towards both Gram-positive and Gram-negative bacteria has also been observed as well as that ILs possess anti-viral activity. In this respect, it has been found, for example, that some ILs can stop the replication cycle of the human immunodeficiency virus (HIV) by inhibiting the function of HIV-integrase, which is an important enzyme in the virus replication cycle (Maddali et al. 2011).

The overall picture that emerges is quite promising: the selective abilities of ILs, together with their extreme flexibility in design, make them potential candidates against cancers, bacteria, and viruses. However, even though there are a relevant number of studies on the “toxic ability” of ILs, only very few studies focus on the mechanisms/mode of action (MoA) of these organic electrolytes (Kumari et al. 2020a). The knowledge of such mechanisms is very important to facilitate and tune the development of applications of ILs in bio-nanotechnology and bio-nanomedicine. In this context, several biophysical studies on the interaction between ILs and biomolecules/biosystems have been performed in the last 10 years showing, for example, the ability of some ILs to stabilize proteins and enzymes, to control protein amyloidogenesis, to diffuse into biomembranes, and to bioprocess and store DNA at room temperature (Benedetto and Ballone 2016a, b; Benedetto and Galla 2017, 2018). These biophysics studies of ILs highlighted their remarkable interactions with bio-molecules, biosystems, and biomembranes in particular, pointing to promising applications in pharmacology, biomedicine, and bio-nanotechnology (Passos et al. 2016; Egorova et al. 2017; Forero-Martinez et al. 2022).

Recent studies have highlighted the high affinity of ILs with phospholipid bilayers (Benedetto 2017; Wang et al. 2018) and motivated several chemical-physical investigations aimed at determining the microscopic mechanisms behind their interaction. This is the focus of this review work.

These studies have been carried out on a variety of different phospholipid bilayer systems ranging from 1-lipid component to 5-lipids component systems, mimicking plasma membranes, and also on a number of cell-extracted membranes. These studies have been carried out at different chemical-physical conditions, approaching the salt content and pH of the cytosol of living cells, and novel applications have also been explored. Several approaches have been used including atomic force microscopy (AFM), small-angle neutron scattering (SANS), small-angle X-ray scattering (SAXS), dynamic light scattering (DLS), zeta potential measurements, differential scanning calorimetry (DSC), quartz crystal microbalance (QCM), solid-state nuclear magnetic resonance (NMR) spectroscopy, confocal fluorescence optical microscopy, and classical molecular dynamics (MD) computer simulations. Overall, the affinity between ILs and phospholipid bilayers results from the fine balance of different interactions including Coulombic and dispersion forces, hydrophilic – hydrophobic competition, and is affected by the structure and the dynamics of hydrogen bonds. The aim of this review work is to provide an *up-to-date* overview of this research field, with the aim to boost it and expand its perimeter towards novel high-impact applications in pharmacology, drug delivery, biomedicine, and bio-nanotechnology in general.

Since my last review of this field (Benedetto 2017), new researcher groups/labs have joined the field and a good number of new studies have been published. The aim of this review is to report on all of them — still a feasible task — allowing any interested reader to gain a much complete as possible overview of the field of “ILs and lipid bilayers”. With this aim in mind, the review focuses on IL-lipid bilayer studies/findings only (and deeply). The review is organized in paragraphs, each of them dedicated to the work carried out by a single and extended research group/lab active in the field. In my opinion, this way of presenting the results of our scientific community has a number of advantages for the reader, for example, allowing to focus on specific techniques, as usually used consistently by the same research group, and following the evolution of the research in time. However, it carries also some disadvantages, including the attribution to a work authored by more than one principal investigator to a single group/lab. In doing so, I have tried my best — I grouped the collaborative works and allocated to one group/lab based on topics, main authors, and in the interest of giving a coherent picture to the reader. I apology in advance for any potential mistake I could have made. Moreover, this way of presenting the work carried out so far in the field, make also easier to present the contribution of my lab in a dedicated paragraph, this because this review work constitutes the “invited award review” for the “2023 Michèle Auger Award” that I was honored to receive earlier this year.

## Benedetto – My contribution to the field

**Fig. 2:**
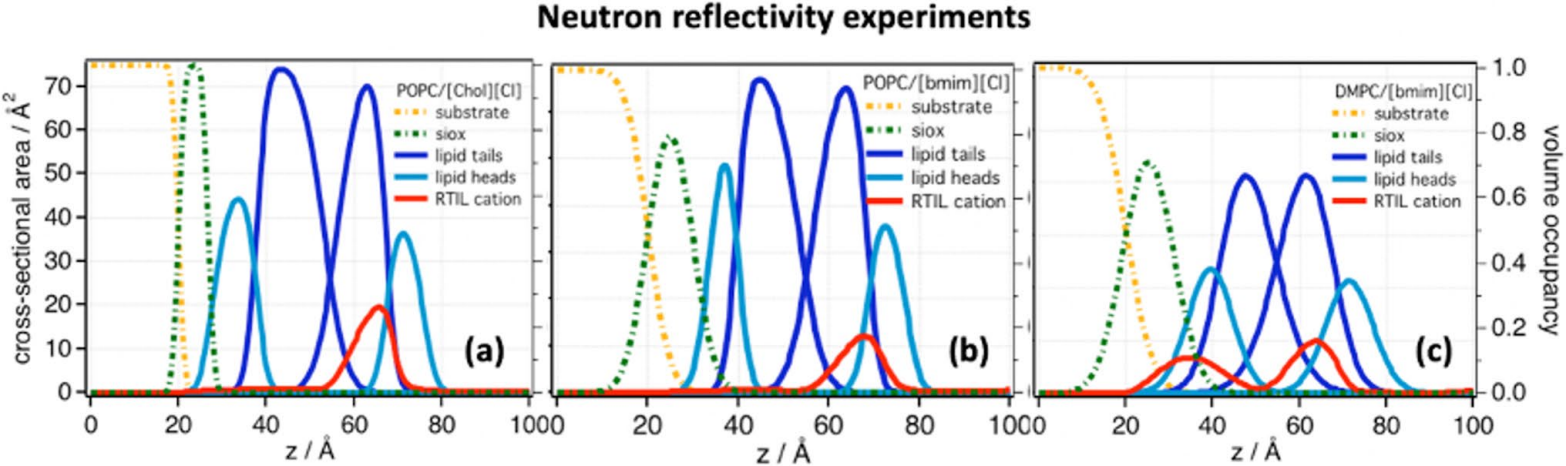
Density distribution profiles as a function of height *z* from the surface of the substrate obtained by fitting the neutron reflectivity (NR) data (Benedetto et al. 2014b). NR has allowed to model each single phospholipid bilayer with four different density distributions accounting for (i) inner lipid heads layer (cyan), (ii) inner lipid tail layer (blue), (iii) outer lipid tail layer (blue), (iv) outer lipid heads layer (cyan), and also (v) the density distribution of the IL-cations (red). Three cases are here reported where two different phospholipid bilayers interact with aqueous solutions of two different ILs at 0.5 M: **a** POPC and [Chol][Cl], **b** POPC and [bmim][Cl], and **c** DMPC and [bmim][Cl]. Figure reproduced with permission from the publisher

My co-workers and I have contributed with several studies to the field of “ILs and lipid bilayers.” Our major approaches include neutron scattering (reflectometry, SANS, spin-echo, elastic and quasi-elastic scattering), AFM for both morphology and mechanics, and MD computer simulations, which we combine with a number of complementary techniques such as DLS. Our first contribution to the field was published in 2014 — almost 10 years ago — and it is a neutron reflectometry study probing the effect of sub-molar concentrations of 1-butyl-3-methylimidazolium chloride ([bmim][Cl]) and choline chloride ([Chol][Cl]) on the structure of 1-palmitoyl-2-oleoyl-glycero-3-phosphocholine (POPC) and 1,2-Dimyristoyl-sn-glycero-3-phosphocholine (DMPC) supported lipid bilayers (Benedetto et al. 2014b). We have shown that, upon incubation of the ILs at the water-bilayer interface, (i) the lipid bilayer shrinks by about 1 Å, (ii) the IL-cations diffuse in the lipid region positioning between the heads and tails of the lipids of the outer lipid leaflet, with the exception of [bmim][Cl] that diffuses into the inner leaflet of DMPC lipid bilayers (Fig. 2), (iii) the amount of ILs partitioning in the lipid region depends on the lipid choice, ranging from 5% of the bilayer volume in POPC to 10% in DMPC, and (iv) rinsing the bilayer in pure water does not remove completely the absorbed ILs.

**Fig. 3:**
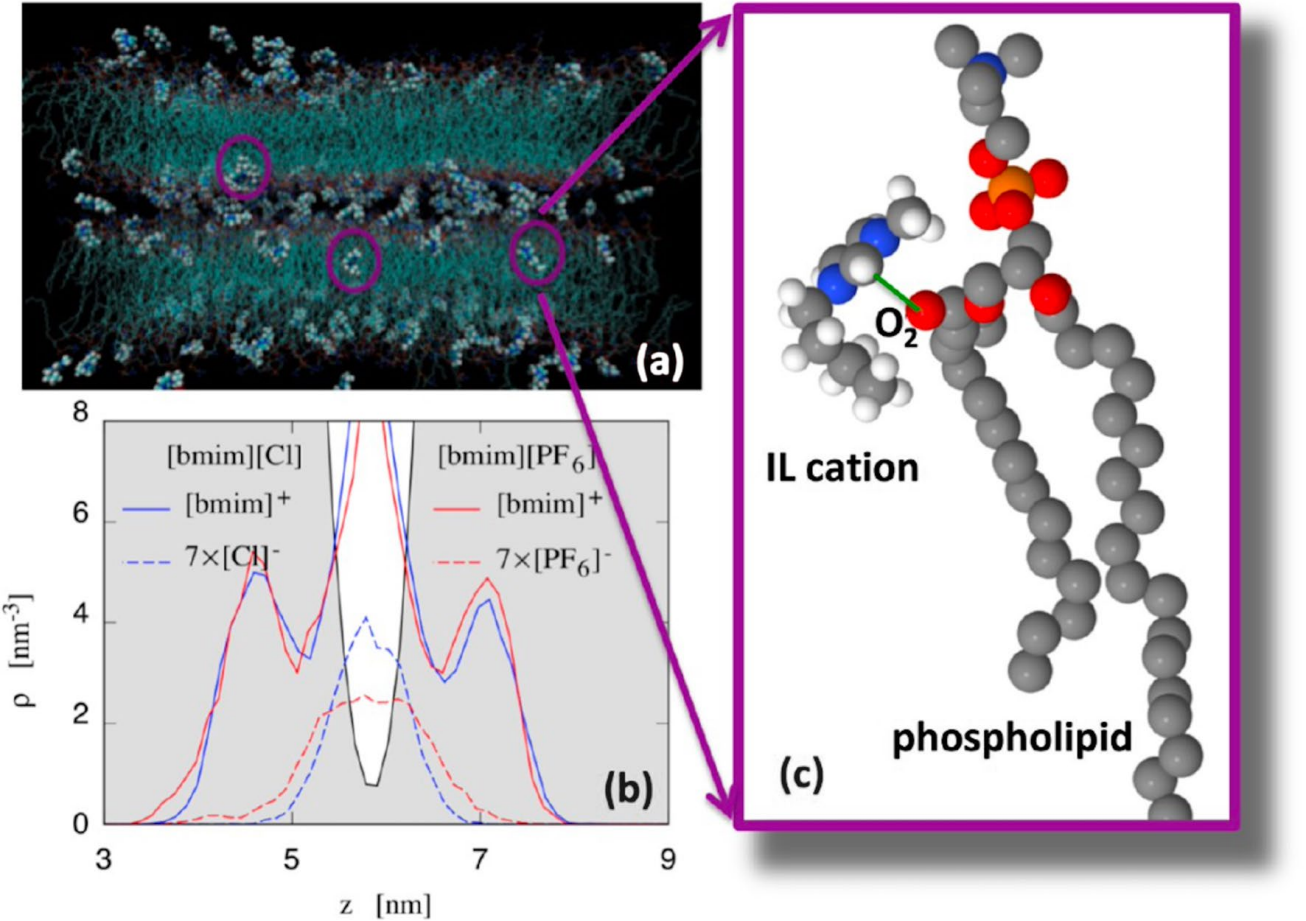
**a** Simulation snapshot showing [bmim]^+^ cations diffused into POPC bilayers. **b** Comparison of the density distribution of cations and anions in [bmim][Cl] and [bmim][PF_6_]. The light shaded area corresponds to the density profile of lipid atoms. **c** Representative configuration of a cation in close contact with a POPC molecule. Adapted from (Benedetto et al. 2015) and reproduced with permission from the publisher

**Fig. 4:**
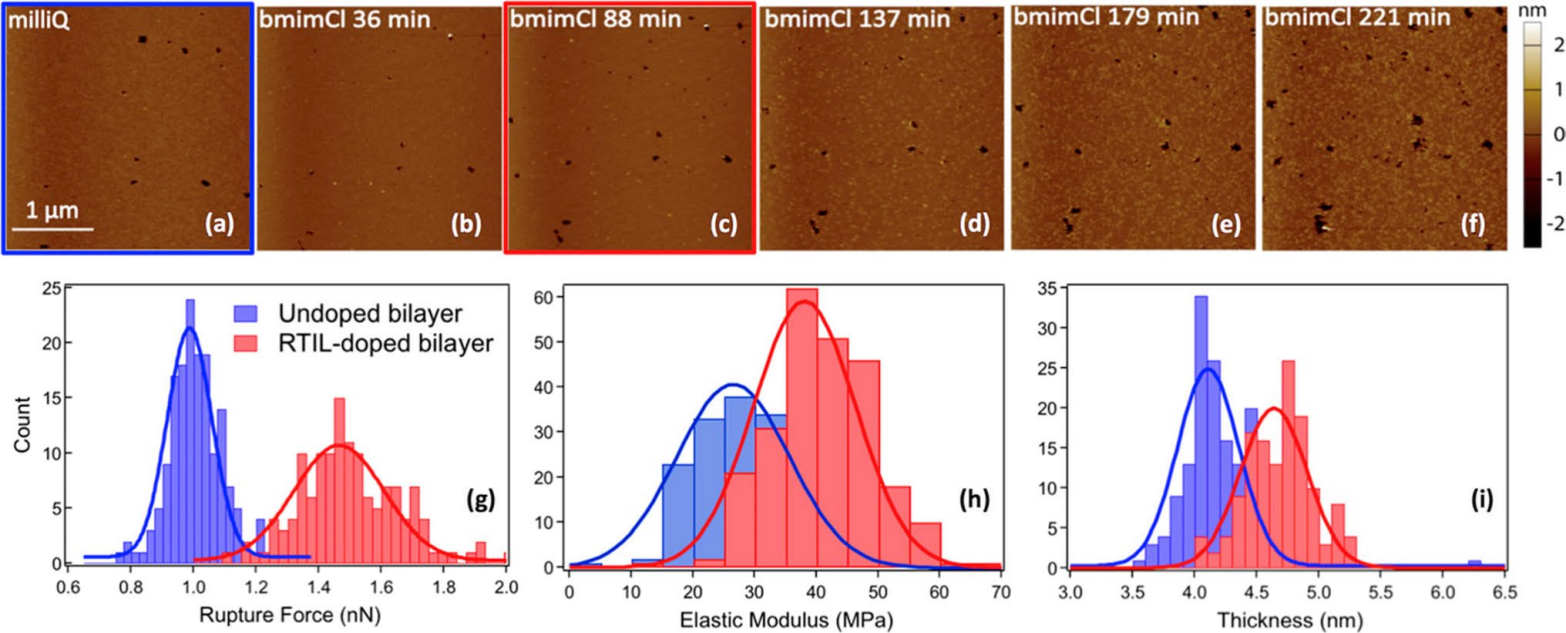
**a–f** AFM height images of a uniform POPC bilayer imaged in milliQ and IL/water solution up to 4 h of incubation. **g** Rupture forces, **h** elastic moduli, and **i** thicknesses for the neat (in blue) and IL-treated lipid bilayer after about 1.5 h of incubation (in red). The rupture force and the elastic modulus increase by about 50% upon the IL absorption into the lipid phase. Taken from (Rotella et al. 2018) and reproduced with permission from the publisher

With the aim to gain a detailed picture of the IL-lipid bilayer interaction, we carried out an extensive classical MD simulation study on the interaction between POPC lipid bilayers with water solutions of [bmim][Cl] and (1-butyl-3-methylimidazolium hexafluorophosphate) [bmim][PF_6_] (Benedetto et al. 2015). The study confirmed the fast incorporation of the IL-cations into the lipid phase, which is driven by the Coulomb attraction between the IL-cation with one of the most electronegative oxygens in the POPC headgroup, and then stabilized by the dispersion forces between the neutral hydrocarbon tails of POPC and [bmim]^+^ (Fig. 3). The location of the IL-cation, absorbed into the lipid phase, resulted in agreement with the above-mentioned neutrons reflectivity study, and this, in turn, supported the validity of the simulation results from which a number of other observables have been computed, including structure and dynamics of the hydrogen bonds, diffusion coefficients of lipids and IL-cations, lipid/water interfacial tension, and bilayer bending rigidity. On this latter, the simulation’s trajectories shown that the presence of the IL-cations in the lipid phase decreases the bending rigidity of the lipid bilayer. With the aim to probe/check this experimentally, we carried out a set of AFM studies on supported POPC lipid bilayers in interaction with a water solution of the imidazolium-based IL [bmim][Cl] (Rotella et al. 2018). We have shown that, upon incubation in the IL/water solution, bilayer elastic modulus and breakthrough force both increase, while the overall stability of the bilayer is kept (Fig. 4). Interestingly, we also observed a “healing effect” of the IL, once added to a defected-lipid bilayers, the IL was leading to an almost defect-free bilayer by closing the pores/defects present. In the same year, in a feature-type work, we published the very-first very-preliminary AFM result on the effect of ILs on live cell morphology and elasticity, along with a set of other very-preliminary results, including one on the ability of magnetic fields to drive (into targets) magnetic ILs-containing liposomes (Benedetto and Ballone 2018).

**Fig. 5:**
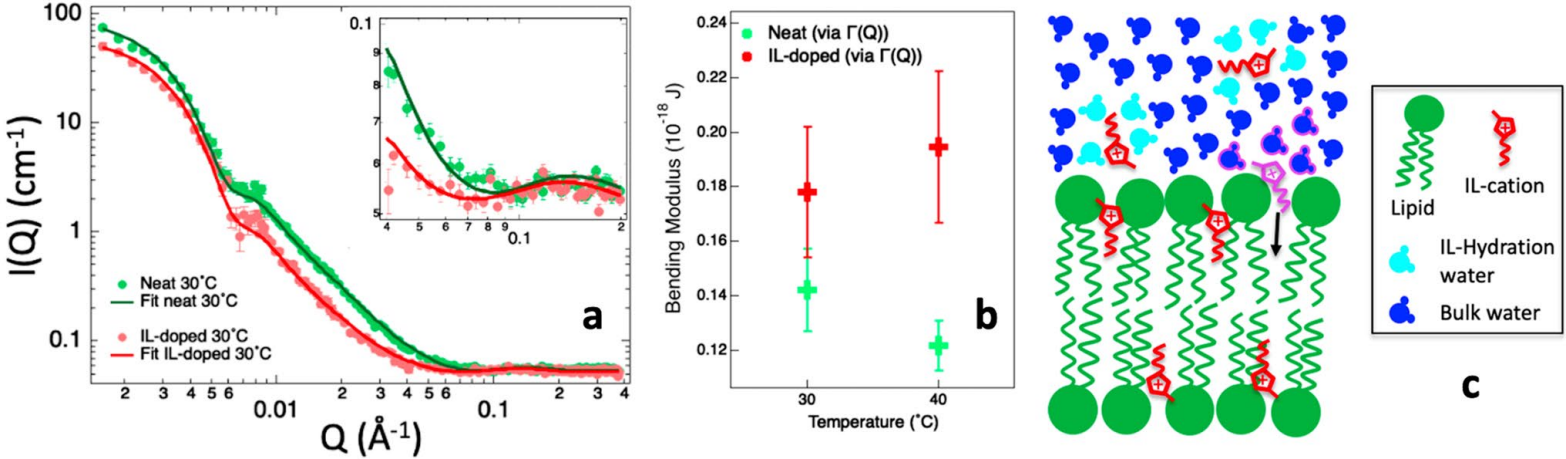
**a** SANS data (circle) collected on neat (green) and [bmim][Cl]-containing (red) DMPC lipid vesicles at 30 °C together with the fitting curves obtained using the polydisperse core 3-shell model, with error bars representing standard deviations. **b** Bending modulus for neat (green) and [bmim][Cl]-containing (red) DMPC lipid vesicles obtained from the neutron spin-echo intermediate scattering functions. **c** Sketch of the IL hydration water entropy-driven absorption mechanism proposed to explain the observed increase on the number of IL cations absorbed in the lipid fluid phase with increasing temperature. When the pink-colored IL-cation diffuses into the lipid bilayer, its pink-circled hydration water molecules get released into the bulk solvent. The entropy variation associated with the release of these water molecules is positive and could be the driving force governing the IL absorption. Taken from (Kumari et al. 2021b) and (Benedetto and Kelley 2022), and reproduced with permission from the publishers

In a follow-up study (Kumari et al. 2020b), we looked at the effect of IL chain length on the elasticity and breakthrough force of IL-containing 1,2-dioleoyl-sn-glycero-3-phosphocholine (DOPC) lipid bilayers. It turned out that the ILs reduce both elasticity and breakthrough force of the bilayer, with the stronger effect observed for the longer-chain IL, while keeping the integrity of the bilayer. In the same work, we correlated this lipid bilayer-effect to the ability of the ILs to alter/control live cell migration — our working hypothesis is that sub-toxic concentrations of ILs can enhance cell migration by reducing the elasticity of the cellular lipid membrane. By having compared the results of the two AFM studies presented above, it turned out that in some cases the addition of ILs can lead to stiffer lipid membranes and in other cases to softer lipid membranes, depending on the ILs and lipids choice. In this context, we looked at the effect of one of these ILs, [bmim][Cl], on the elasticity of a lipid bilayer made by a different lipid, i.e., DMPC (Kumari et al. 2021b). With SANS, we accessed the IL partitioning between the lipid and the aqueous phases, showing that approximately 5 IL-cations diffuse in the lipid phase for each 11 lipids at 30° C when the bilayer is in its fluid phase (Fig. 5a). Then the effect of the absorbed IL-cations on the bending elasticity was investigated by means of neutron spin-echo spectroscopy, showing an increase in the bilayer’s bending modulus upon incorporation of the IL-cations (Fig. 5b). In a follow-up study (Benedetto and Kelley 2022), we extended our SANS investigation to look at the IL partitioning also in the gel and ripple phases. We have shown that, also in these lipid phases, the IL-cations can diffuse in the lipid region at concentrations that are only slightly smaller if compared to the fluid lipid phase case. In this context, we commented on the fact that one the key driving forces behind IL-cation absorption might be of entropic nature, being the entropy gain coming from the IL hydration water once the cation is absorbed (Fig. 5c).

**Fig. 6:**
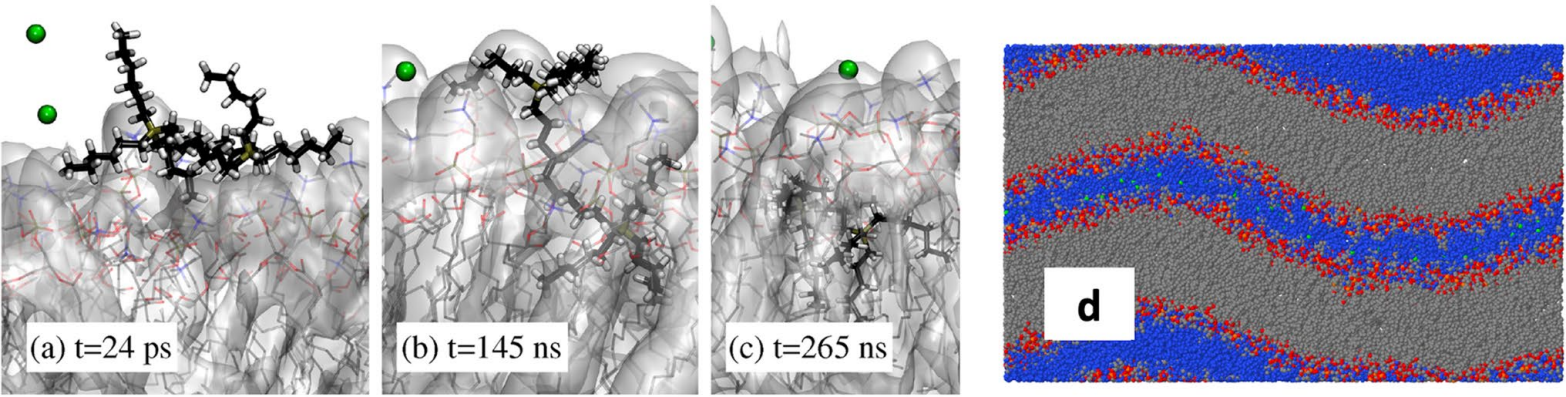
**a–c** Sequence of snapshots from the simulation illustrating the successive stages of one phosphonium di-cation entering the lipid bilayer. **a** The di-cation is absorbed at the water/lipid interface, **b** one of the di-cations heads entered the lipid phase, and **c** the whole di-cation is absorbed in the lipid phase. **d** Cross-section in the xz-plane of the POPC + 120 [P_6666_][Cl] snapshot taken after 3.5 µs of equilibration showing the bilayer in a ripple-like phase. Taken from (Pillai et al. 2022b) and reproduced with permission from the publishers

In our (so far) last contribution to the field (Pillai et al. 2022b), we employed classical MD simulations to look at the absorption of phosphonium mono- and di-cations into a POPC lipid bilayer. We have found that the absorption occurs during a time-scale of several microseconds and is irreversible, with the counterions [Cl]^−^ remaining dissolved in water and giving origin to a strong electrostatic double layer at the water/bilayer interface. The absorption of the phosphonium IL-cations alters several bilayer properties, including viscosity and lipid diffusion. The major difference between mono- and di-cations is observed in their absorption kinetics, which is about two times faster for the mono-cation and displays two steps for the di-cation corresponding (i) first to the absorption of the first phosphonium head, (ii) followed by the more activated absorption of the second phosphonium head (Fig. 6a–c). Interestingly, at high IL concentrations, the lipid bilayer changes its phase from fluid planar to ripple (Fig. 6d).

## Galla & Glorius

**Fig. 7:**
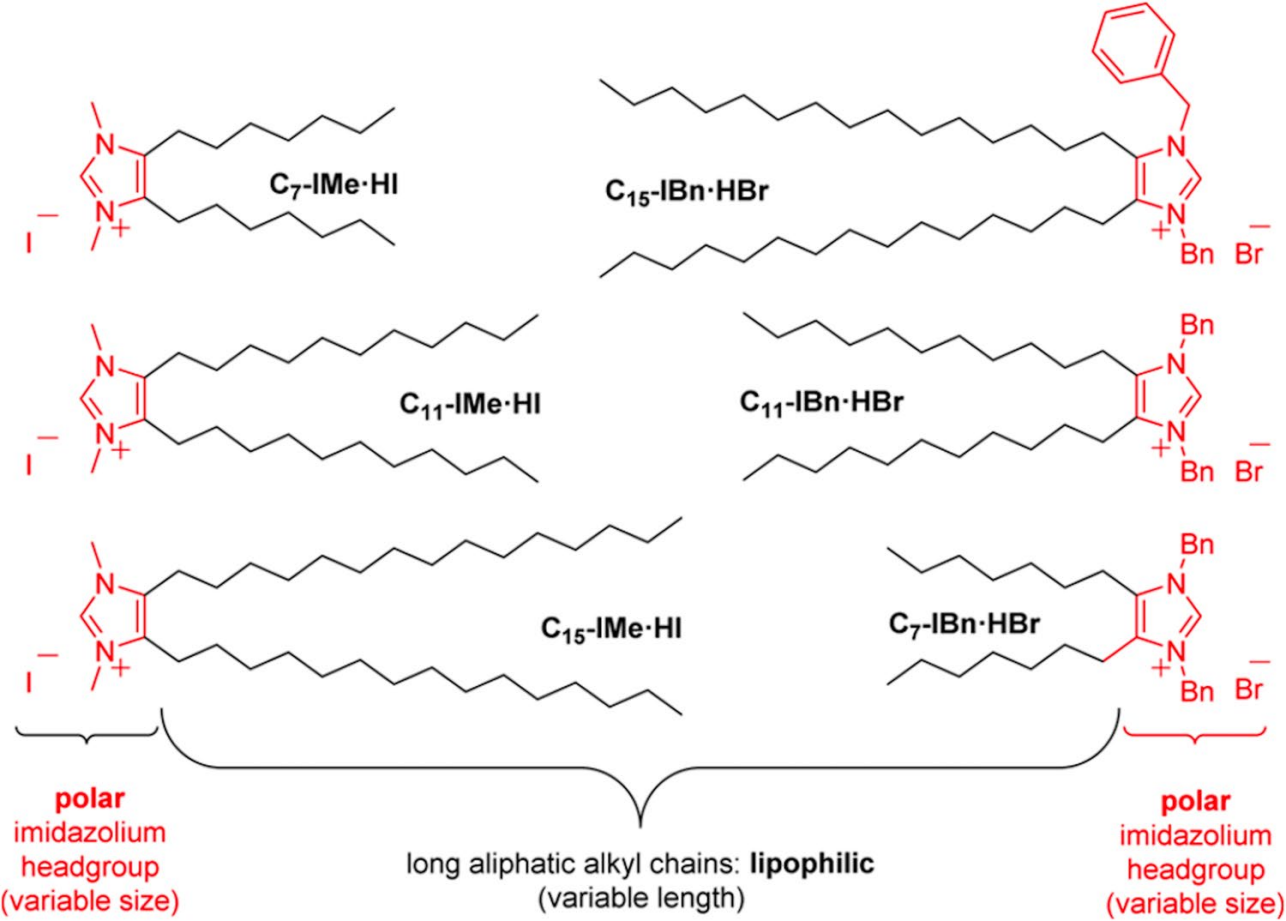
Some examples of the double-tail imidazolium-based lipid-mimicking IL synthesized by Galla and co-workers. Taken from (Drüker et al. 2017) and reproduced with permission from the publisher

**Fig. 8:**
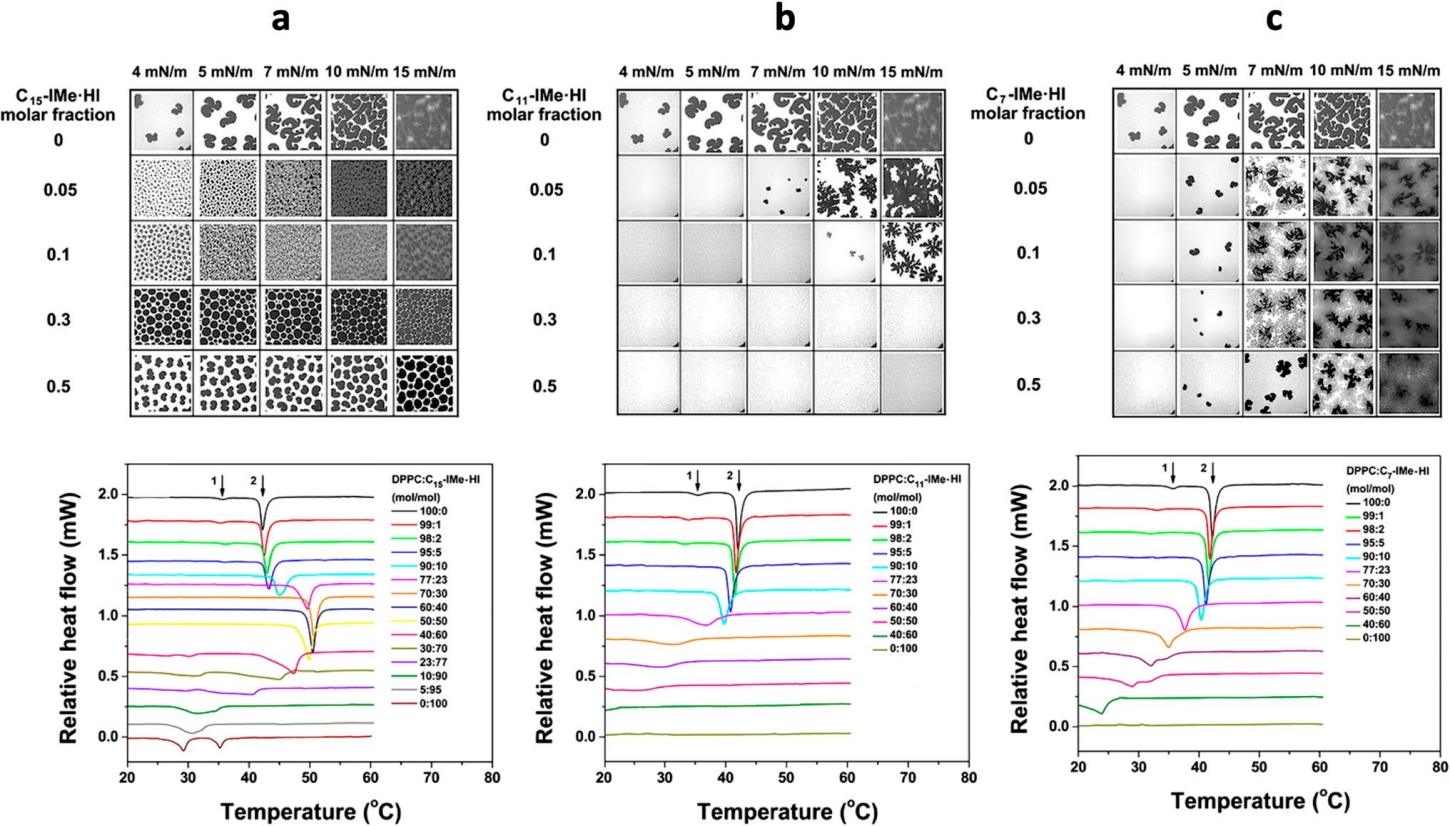
Epifluorescence images of the mixed monolayers of DPPC at the air–water interface at room temperature (top row) and differential scanning calorimetry endotherms of large unilamellar vesicles of DPPC in pure water (bottom row) with different molar fractions of **a** C_15_-Ime⋅HI, **b** C_11_-Ime⋅HI, **c** C_7_-Ime⋅HI. Taken from (Wang et al. 2016) and reproduced with permission from the publisher

Galla and co-workers have contributed with several novel studies to the field, and have also explored a number of intriguing potential applications. Their major approaches include confocal fluorescence microscopy, fluorescence microscopy and spectroscopy, DSC, film balance, and QCM. In one of their first work (Wang et al. 2015a) — to be best of my knowledge their first contribution to the field — they have synthesized a new class of imidazolium-based ionic liquids, bearing two alkyl chains in the backbone of the imidazolium core, and investigated their effect, as function of the chain length, on (i) DPPC monolayers, by means of film balance measurements, and on (ii) POPC:POPS (4:1 molar ratio) liposomes tethered on a solid substrate, by means of QCM. The critical micellar concentration (CMC) of the ILs in aqueous solutions was determined. This is defined as the concentration at which the ILs dispersed in water start to self-assemble in supermolecular structures such as micelles or vesicles. It turned out that the matching/mismatching between the chain length of lipids and ILs play a key role in their mutual interaction, along with their concentration in respect to the CMC values. They have shown that (i) the longer-chain IL (which is the one matching the lipid chain length) nicely fuses/intercalates within the lipid membrane without considerably affecting its stability, (ii) the medium-chain IL partially intercalates with the lipid membrane, but the hydrophobic mismatch already destabilizes the membrane, and (iii) the shorter-chain IL has a very little interaction with the lipid membrane but, surprisingly, it has the highest permeation activity. A link between lipid membrane activity (intercalation vs penetration) and the cytotoxicity of these ILs has been also proposed. The CMC and surface tension of a number of these double-tail lipid-mimicking ILs have been obtained (Wang et al. 2015b). In a follow-up study (Wang et al. 2016), Galla and co-workers looked at the effect of these family of double-tail imidazolium-based ILs on DPPC monolayer and bilayers by means of a number of different approaches, including film balance measurement coupled with epifluorescence microscopy and DSC (Fig. 7). The epifluorescence imaging study shown the formation of different lipid-IL domains (Fig. 8, top row). The DSC investigations shown that the addition of IL can shift the lipid main transition temperature by about 10 degrees, depending on the length of the hydrocarbon chain and the IL concentration (Fig. 8, bottom row). Overall, it turned out the longer-chain IL (C_15_-Ime⋅HI) forms a thermodynamically-favored kinetically-reversible Langmuir monolayer with DPPC and induces a stiffening of the DPPC bilayer. The incorporation of the medium-chain IL (C_11_-Ime⋅HI), on the contrary, causes the formation of a thermodynamically-unstable kinetically-irreversible Langmuir-Gibbs monolayer with DPPC and disordered DPPC liposomes. The shorter-chain IL (C_7_-Ime⋅HI) displays negligible membrane activity instead.

**Fig. 9:**
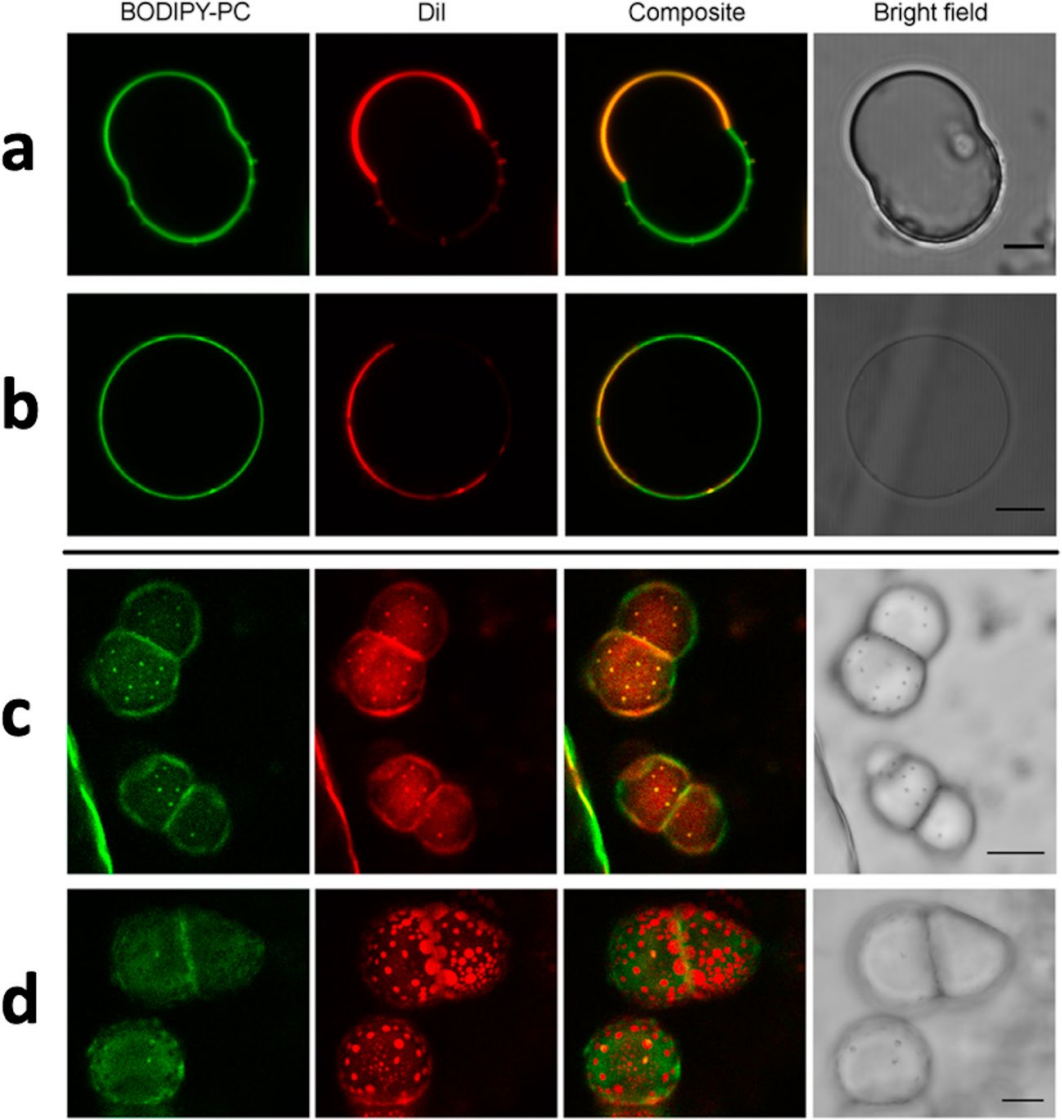
Bilayer domain fluidization in the presence of 10% of the double-tail imidazolium-based IL C_15_-Ime⋅HI. **a** Giant unilamellar vesicles (GUV) of DOPC/PSM/Chol (33:33:33) at T = 27.3° C, scale 5 µm. **b** GUVs of DOPC/PSM/Chol/IL (33:23:33:10) at T = 27.3° C, scale 10 µm. Note the difference in line tension between domains. **c** GUVs of DOPC/SSM/Chol (33:33:33) at T = 38.0° C, scale 20 µm. **d** GUVs of DOPC/SSM/Chol/IL (33:23:33:10) at T = 38.0° C, scale 20 µm. Note the prominent fluidization of small bulged domains to flat large domains with enhanced dye specificity upon IL addition. Taken from (Drüker et al. 2017) and reproduced with permission from the publisher

**Fig. 10:**
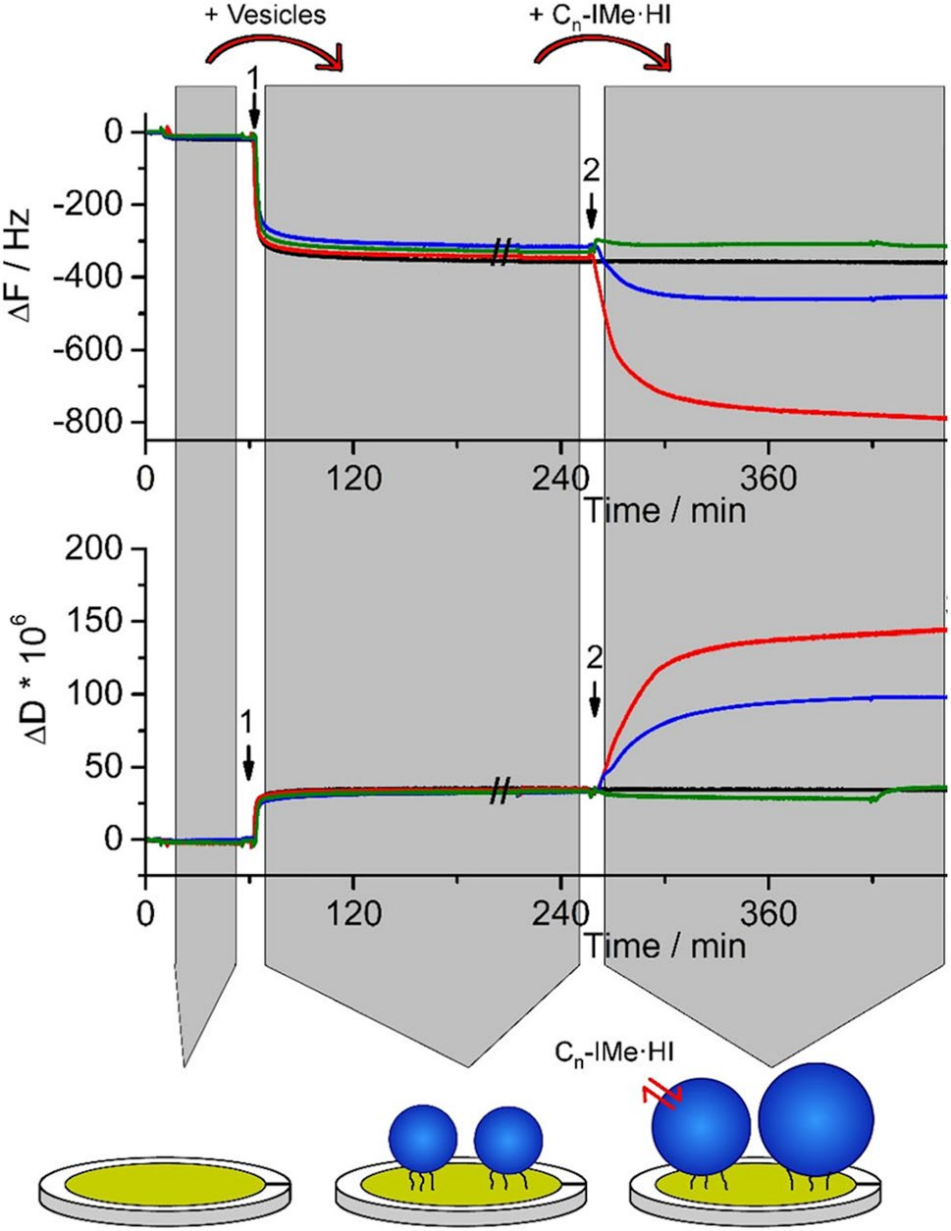
Interactions of the double-tail imidazolium-based ILs C_n_-IMe⋅HI with POPC:POPS (4:1) large unilamellar vesicles (*d* = 200 nm) probed by quartz crystal microbalance. The large unilamellar vesicles were surface-tethered by biotin-streptavidin linkers on a self-assembled monolayer coated sensor surface (arrows 1). Following equilibration with tris-buffered-saline, pH 7.4, suspensions of 0.1 mM ILs were incubated, which induced diverse membrane interactions (arrow 2). The longer-chain and the medium-chain ILs (*n* = 15,11), in red and blue respectively, lead to an increase in surface associated mass, indicated by ∆F and an increased viscoelasticity of the associated layers indicated by *∆D*. On the contrary, the shorter-chain IL (*n* = 7, in green) did not alter much the lipid membrane. Taken from (Wang et al. 2018) and reproduced with permission from the publisher

The role played by different headgroups have also been investigated, showing that smaller and hydrophilic headgroups induce membrane stiffening, while bulkier and more hydrophobic headgroups make the membrane more fluid (Rühling et al. 2017). In an additional study (Drüker et al. 2017), by means of fluorescent bilayer probes, Galla and co-workers looked at how these double-tail ILs affect membrane hydration properties and domain fluidization (Fig. 9). The study has been carried out on established model membranes in a buffered aqueous environment resembling the salt content and pH of the cytosol of living cells. The combination of IL with different chain length and different headgroups resulted in a variety of different effects ranging from “penetration without disruption of the membrane” to “disintegration and lysing of the membrane.” In 2018, Galla and co-workers published a review paper focused on the effect of their lipid-like double-tail imidazolium-based ILs (Wang et al. 2018). In this work they nicely summarized the use of QCM to probe IL-membrane interaction (Fig. 10), and the penetration, disruption and fusing effects of ILs they observed on lipid membrane.

**Fig. 11:**
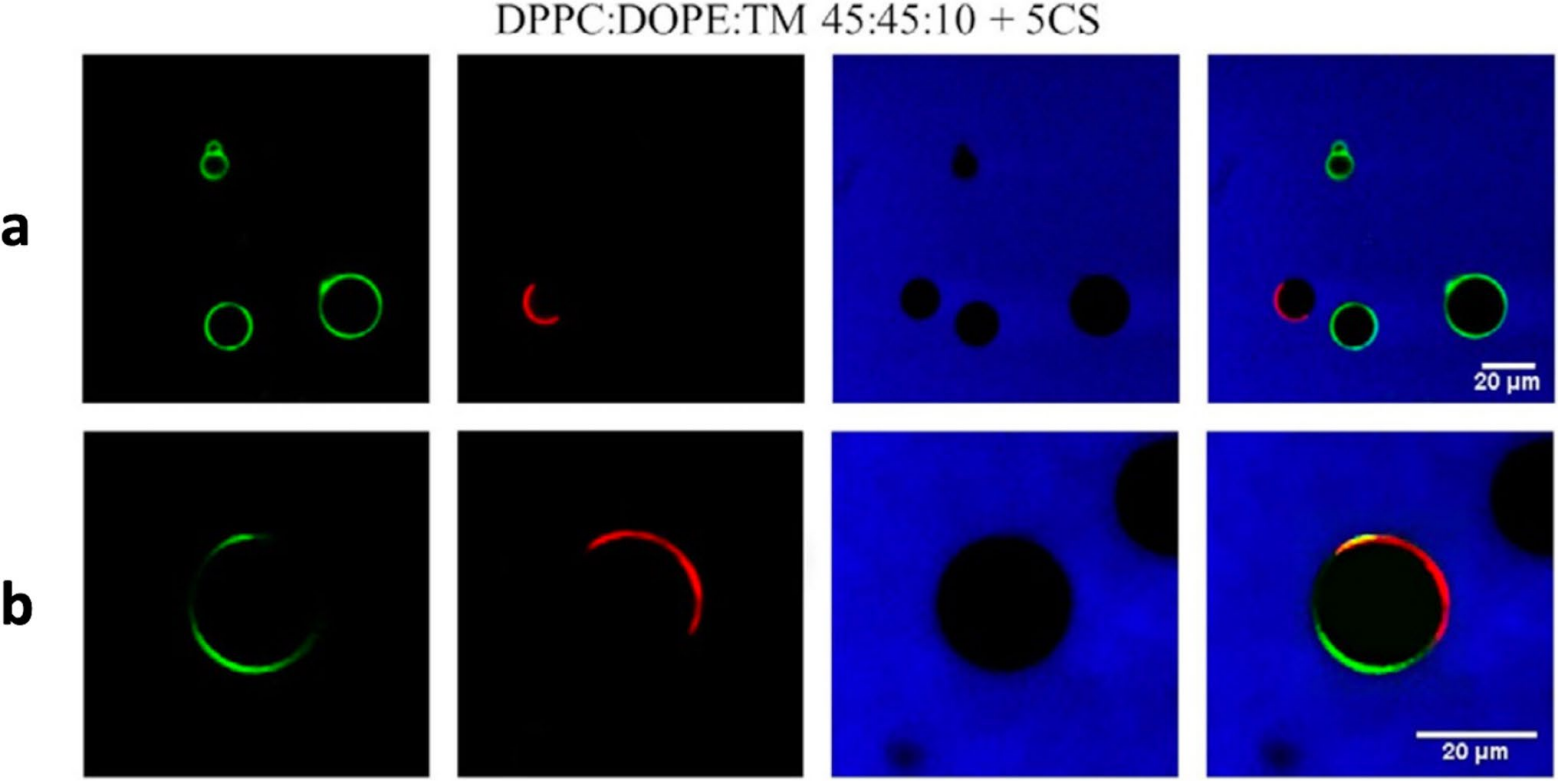
Confocal fluorescence microscopy cross-sectional images of the IMeNMe_3_ IL vesicle fusion experiment taken from (Paulisch et al. 2020). First column: GUVs labelled by NBD-DHPE; second column: GUVs labelled by N-rhodamine-DHPE; Third column: Buffer marked with Atto-647; fourth column: Combined image. **a** GUVs without the IL, and **b** GUVs with the IL. The scale bar of the images corresponds to 20 µm. Reproduced with permission from the publisher

In two follow-up works (Paulisch et al. 2020; Bornemann et al 2020), Galla and co-workers investigated the effect of their double-tail ILs on lipid bilayers of increasing complexity considering: the 1-component DPPC bilayer, the 3-component DPPC:DOPC:cholesterol (2:1:1) and DPPC:DOPE:Chol (2:1:1) bilayers, and the 5-component DPPC:DPPG:DOPC:DOPG:Chol (45:5:20:5:25) bilayer, this latter served as a refined model of a biological plasma membrane. Fluorescence spectroscopy was used to look at the effect of the ILs on the lateral order and fluidity of the lipid membrane, showing an increase of the liquid-disorder domain order parameter in the 3-component and 5-component lipid bilayers upon incubation in the ILs. AFM was used to look at the change in membrane morphology induced by the ILs, showing a slightly reduction in the height-difference between liquid-order and liquid-disorder domains upon IL incubation, as well as a size reduction for the liquid-disorder domain. Zeta potential measurements allowed to probe the effect of the ILs on the surface charge density of the lipid membranes, showing the absorption of the ILs into the membrane. Confocal fluorescence microscopy was used on giant unilamellar vesicles, and provided a number of interesting findings, including the fusogenic properties/character of one of the ILs in use. In this context, (i) cargo lipid vesicles of DPPC:DOPE (1:1) containing 10 mol% of the IL and a green fluorophore, and (ii) target lipid vesicles of the 5-component DPPC:DPPG:DOPC:DOPG:Chol (45:5:20:5:25) mix labelled with a red fluorophore were prepared and then mixed together. It turned out that without the IL, no mixing of the green and red fluorophores was observed (Fig. 11a); on the contrary, the presence of the IL was able to induce the mixing (Fig. 11b) showing the fusogenic ability of the IL. On the side, an interesting addition brought into the picture by this team is the synthesis, characterization and applications of a class of versatile and clickable cholesterol-based imidazolium salt (Rakers et al. 2018; Zheng et al. 2023).

## Ghosh & Sharma

**Fig. 12:**
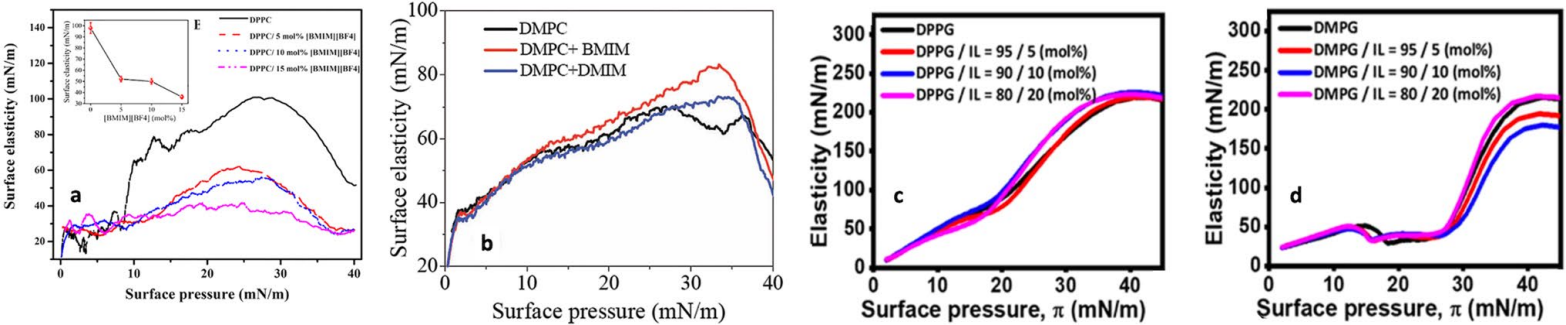
The effect of imidazolium ILs on lipid monolayers’ in-plane elasticity: **a** DPPC lipid monolayer in interaction with [bmim][BF_4_] taken from (Bhattacharya et al. 2017). The change in elasticity at a surface pressure of 30 mN/m is shown in the inset. **b** DMPC lipid monolayer in interaction with [bmim][BF_4_] and [dmim][BF_4_] taken from (Sharma et al. 2017). **c** DPPG lipid monolayer in interaction with [bmim][BF_4_] taken from (Hitaishi et al. 2022). **d** DMPG lipid monolayer in interaction with [bmim][BF_4_] taken from (Hitaishi et al. 2022). Figure reproduced with permission from the publisher

**Fig. 13:**
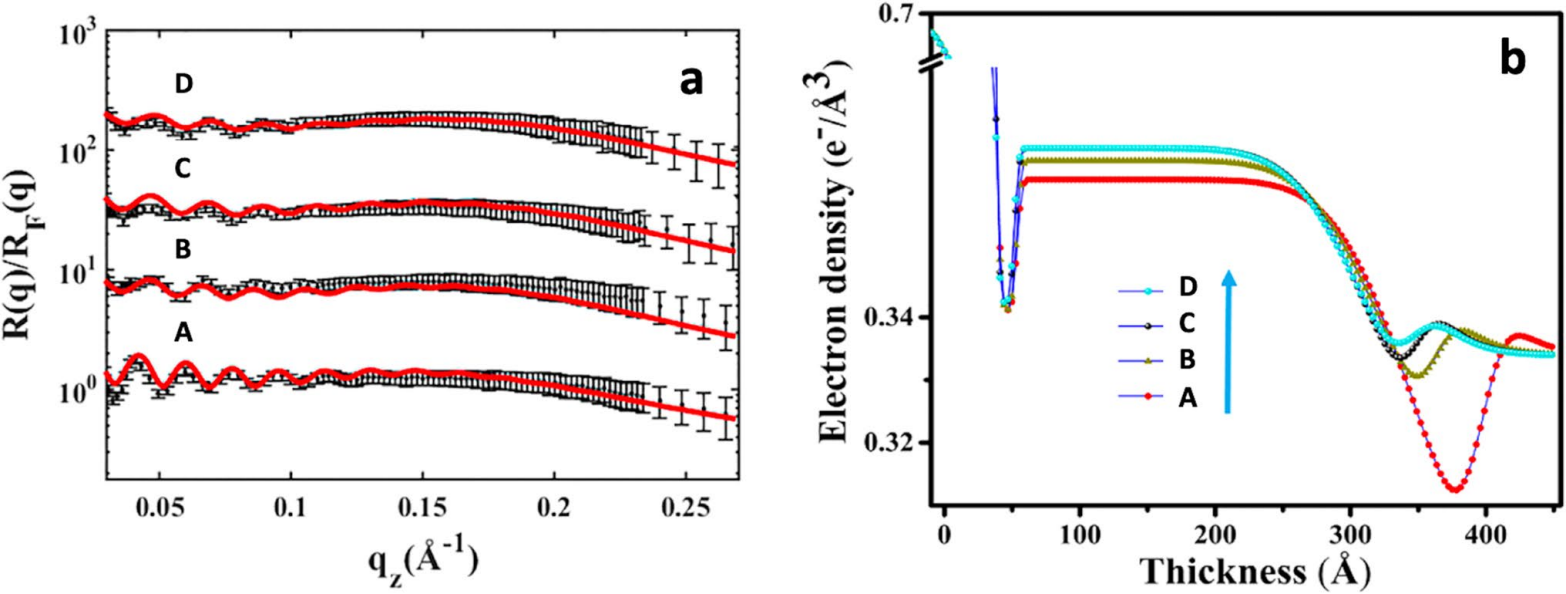
[bmim][BF_4_] on DPPC bilayer taken from (Bhattacharya et al. 2017). **a** X-ray reflectivity measurements carried out in the fluid phase at 48° C on supported lipid bilayer formed on a polymer cushion: (A) neat bilayer, (B) bilayer with 5 mol % of IL, (C) bilayer with 10 mol % of IL, and (D) bilayer with 15 mol % of IL. The curves are shifted vertically for clarity. **b** Corresponding electron density profiles extracted from the fits of the curves in **a**. Figure reproduced with permission from the publisher

Ghosh and co-workers have contributed to the field with several novel studies on the interaction of ILs with lipid monolayers and bilayers. Their major approaches include synchrotron-based reflectivity and SAXS, Langmuir–Blodgett measurements, elastic and quasi-elastic neutron scattering, and DSC. In one of the first work (Bhattacharya et al. 2017) — to be best of my knowledge their first contribution to the field — they have investigated the effect of the imidazolium IL [bmim][BF_4_] on monolayers and bilayers of the zwitterionic lipid 1,2-dipalmitoyl-sn-glycero-3-phosphocholine (DPPC) by means, respectively, of Langmuir-Bloggett apparatus and X-ray reflectivity. They have shown that the IL (i) increases the surface pressure of the lipid monolayer formed at the air–water interface, indicating the penetration of the IL into the lipid phase; (ii) reduces the monolayer in-plane elasticity, from approximately 98 to 36 mN/m at physiological pressure (Fig. 12); (iii) triggers a considerable decrease in the lipid bilayer’s thickness, from approximately 47 to 32 Å in the lipid fluid phase, and a corresponding increase in electron density in both the gel and the fluid thermotropic phases of the lipid bilayer (Fig. 13). The penetration kinetics of the IL into the lipid region was also investigated, showing that it follows a Boltzmann-like equation (Bhattacharya et al. 2018a). In the same work, enthalpy and entropy were computed from the temperature-dependent pressure-area isotherms of the lipid monolayer, showing that the addition of IL destabilizes the lipid phase, which was also confirmed by a shift of the main transition temperature to lower values.

**Fig. 14:**
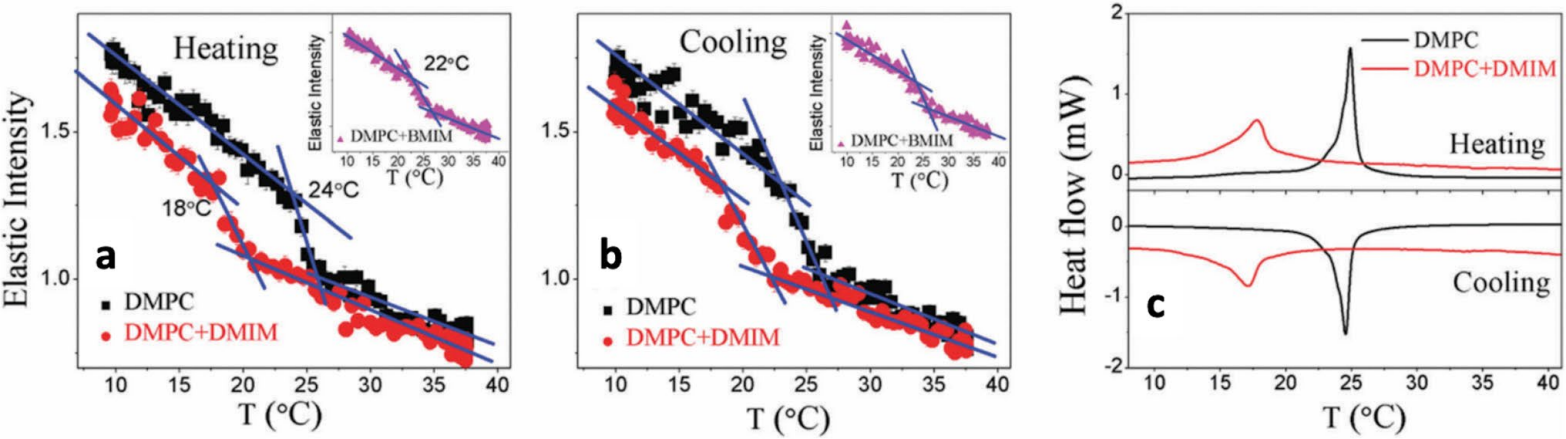
The effect of imidazolium ILs on DMPC phase transition temperature taken from (Sharma et al. 2017). **a** Elastic neutron scattering intensity (Q-averaged) versus temperature for DMPC vesicles neat (black) and treated with [dmim][BF_4_] (red) and [bmim][BF_4_] (inset). **b** Same as in **a** but while cooling. **c** Differential scanning calorimetry thermograph of neat (black) and [dmim][BF_4_]-treated (red) DMPC vesicles. Figure reproduced with permission from the publisher

In 2017, they have published a combined elastic and quasi-elastic neutron scattering study on the effect of [bmim][BF_4_] and 1-decyl-3-methylImidazolium tetrafluroborate ([dmim][BF_4_]) on the nanoscopic dynamics of DMPC vesicles (Sharma et al. 2017). They have shown that due to the presence of the ILs (i) the gel-to-fluid transition temperature of the lipid bilayer moves to lower temperatures (Fig. 14) and (ii) the fluidity of the membrane increases, with the greater effect being observed upon interaction with the longer-tail IL [dmim][BF_4_]. Interestingly, the DMPC monolayer in-plane elasticity, measured with a Langmuir–Blodgett trough apparatus, resulted to increase due to the presence of the two ILs, which is the opposite behavior observed, for example, with DPPC (Fig. 12).

**Fig. 15:**
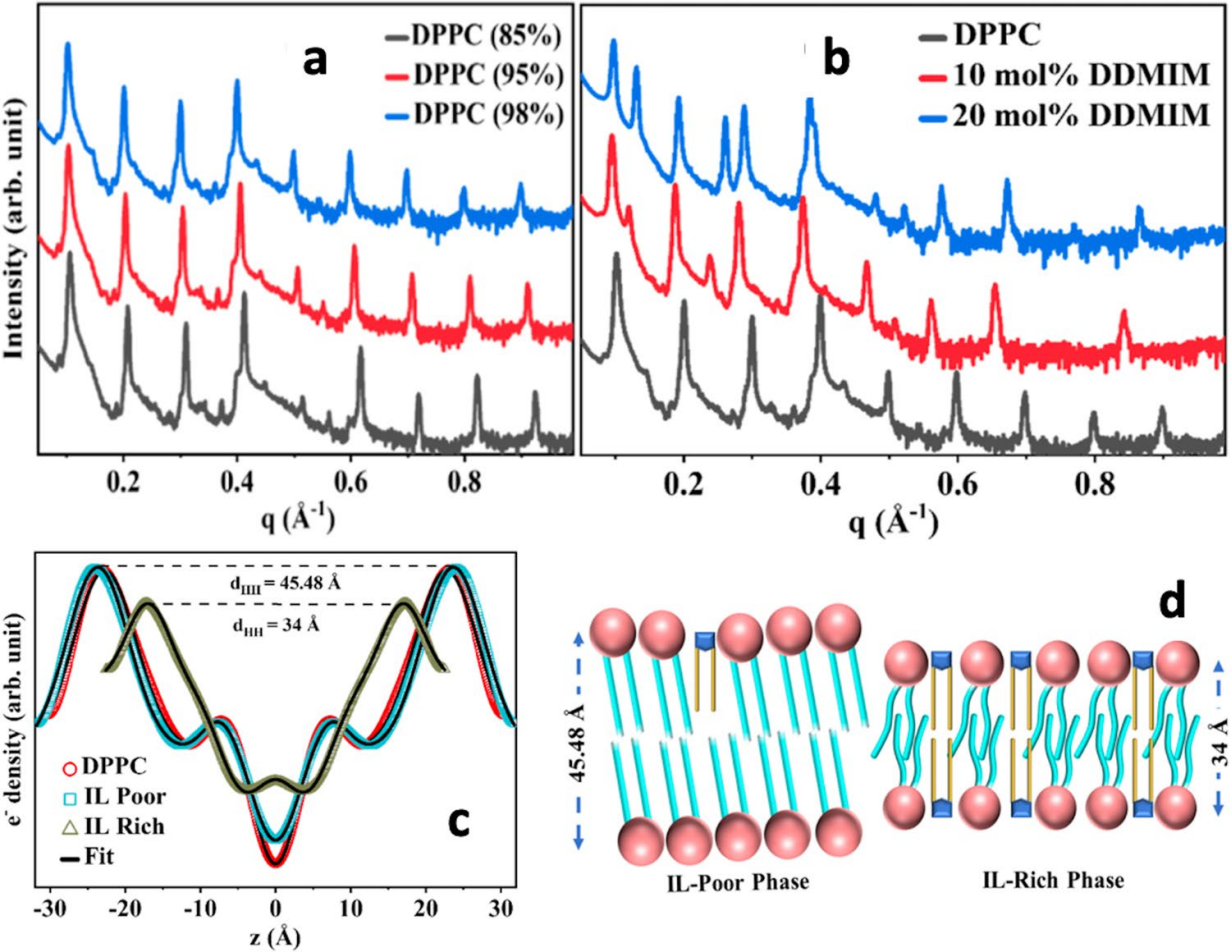
IL induces phase separation and interdigitation in lipid multi-layers from (Gupta et al. 2022). Measured reflectivity profiles of **a** neat and **b** IL-containing oriented stacks of DPPC lipid bilayers at three different RH values. The curves are shifted vertically for clarity. The profiles in **b** show two sets of lamellar reflection peaks, signifying the coexistence of two phases in the IL-containing sample. **c** The associated electron density profiles along the surface normal of the bilayer. d_HH_ is the head-to-head thickness of the bilayers. The IL-poor phase is similar to the neat lipid phase, while the IL-rich phase is interdigitated. **d** Schematic of the IL-rich and IL-poor phases. Figure reproduced with permission from the publisher

In a follow-up study (Bhattacharya et al. 2018b), Ghosh and co-workers have looked at the effect of the presence of negative charged lipids (usually added to mimic bacteria membranes). In this context, monolayers and bilayers of DPPC:DPPS (4:1 molar ratio) have been studied. It turned out that the decrease in the surface elasticity of the mixed DPPC:DPPS monolayer (about 76%) is higher compared to the only zwitterionic DPPC system at similar IL concentrations (about 63%), pointing to the fact that the presence of negatively charged lipids can enhance the IL-membrane interaction. A similar trend was observed on the mixed DPPC:DPPS bilayer — it turned out that already with only 5 mol % of [bmim][BF_4_], the bilayer shrinks to a thickness of about 18 Å, corresponding to a thickness reduction of 57% with respect to the neat bilayer — the thickness reduction observed for the DPPC bilayer at the same condition was of 20% only. In the same work (Bhattacharya et al. 2018b), they looked also at the effect of IL-chain length. The effect of the imidazolium IL [bmim][BF_4_] on monolayers and bilayers of DPPC and DPPC:DPPS lipids was compared with the effect of the imidazolium IL 1-ethyl-3-methylimidazolium tetrafluoroborate ([emim][BF_4_]) that has a shorter hydrocarbon tail. Overall, it resulted that the effects/changes are more prominent in the case of the longer-chain IL. By means of DLS, zeta potential measurements, SAXS, Fourier transform infrared spectroscopy, and polarizing optical microscopy, Ghosh and co-workers have then shown that the above results hold also for lipids arranged in multilamellar vesicles — in this case they looked at the effect of an even longer-tail imidazolium IL, i.e., [dmim][BF_4_] (Mitra et al. 2019). Ghosh and co-workers have also looked at a “double-tail mimicking-lipid IL,” i.e., ^Me^Im(COOH)^Me^(Oleylamine)Iodide. They have shown that this IL behaves as an unsaturated phospholipid, e.g., it is capable of self-assembling and exhibit a thickness and electron density compared to those of unsaturated lipids. Overall, compared to single-hydrocarbon-chained ILs, this double-hydrocarbon-chained IL showed a greater/stronger effect (Mitra et al. 2020). For example, by means of DSC, its effect on the main phase transition temperature of DPPC multilamellar vesicles was studied. It resulted that the presence of the IL reduce the transition temperature from approximately 41° C of the neat system to 38° C at 20 mol % of the IL. With the aim to approach the composition of real biological membranes, Ghosh and co-workers have then looked at the effect of ILs on monolayers and bilayers made of a liver lipid extract (Bakshi et al. 2020). More specifically, a set of imidazolium ILs ([C_n_mim][BF_4_] and [C_n_mim][Cl]), differing in the length of their hydrocarbon chain, have been administered to monolayers and bilayers made of the liver lipid extract PC:PE:PI:lysoPI:Cholesterol:other (42:26:9:1:5:17 weight ratio). The study, carried out on lipid monolayers by means of a Langmuir–Blodgett trough apparatus, has shown that the interaction of the ILs with the lipid layer is energetically favorable and the addition of the ILs reduces the monolayer’s in-plane elasticity. Quasi-elastic neutron scattering experiments, performed on the lipid bilayer in unilamellar vesicle geometry, have shown that the lipid molecules diffuse faster in the IL-treated system. A follow-up more-detailed quasi-elastic neutron scattering study has then published (Sharma et al. 2020), showing that both lateral motion of the lipid within the bilayer leaflet and localized internal motions of the liver extract lipids contribute to the spectrum broadening, i.e., to the enhanced dynamics of the lipids in presence of the IL. Ghosh and co-workers looked then more carefully into the interaction between imidazolium ionic liquids, of different chain length, with monolayer of liver extract lipids (Mitra et al. 2021). By isothermal titration calorimetry they have confirmed that the longer is the IL hydrocarbon tail the stronger is the interaction with the lipid bilayer, finding a stoichiometry parameter of about 2 (lipid molecules for each IL molecule) in the case of their longer-tail IL [dmim][BF_4_] suggesting a permeation of the IL into the core of the lipid bilayer. Moreover, X-ray reflectivity studies, carried out on the lipid monolayer, suggested that low concentrations of the IL shrink the monolayer, while higher concentrations make it thicker. The effect of IL on lipid organization has been further investigated (Gupta et al. 2021). X-ray scattering and AFM have been used to look at the DPPC multi-layer structure upon interaction with ammonium and phosphonium-based ILs with methanesulfonate anion (i.e., TAM, TPM, TAPTS and TPPTS). The obtained data supported a picture in which the ILs trigger the formation of phase-separated domains in the plane of the bilayer — IL-rich domains of interdigitated lipid bilayer and IL-poor domains closely related to the pristine lipid phase. In a follow-up study, the effect of a imidazolium-based double-tail IL was also investigated (Gupta et al. 2022). X-ray reflectivity profiles of oriented stacks of neat and IL-treated DPPC bilayers suggested, also in this case, the formation of IL-rich domains of interdigitated lipids and IL-poor domains, this latter very similar to the neat condition (Fig. 15).

**Fig. 16:**
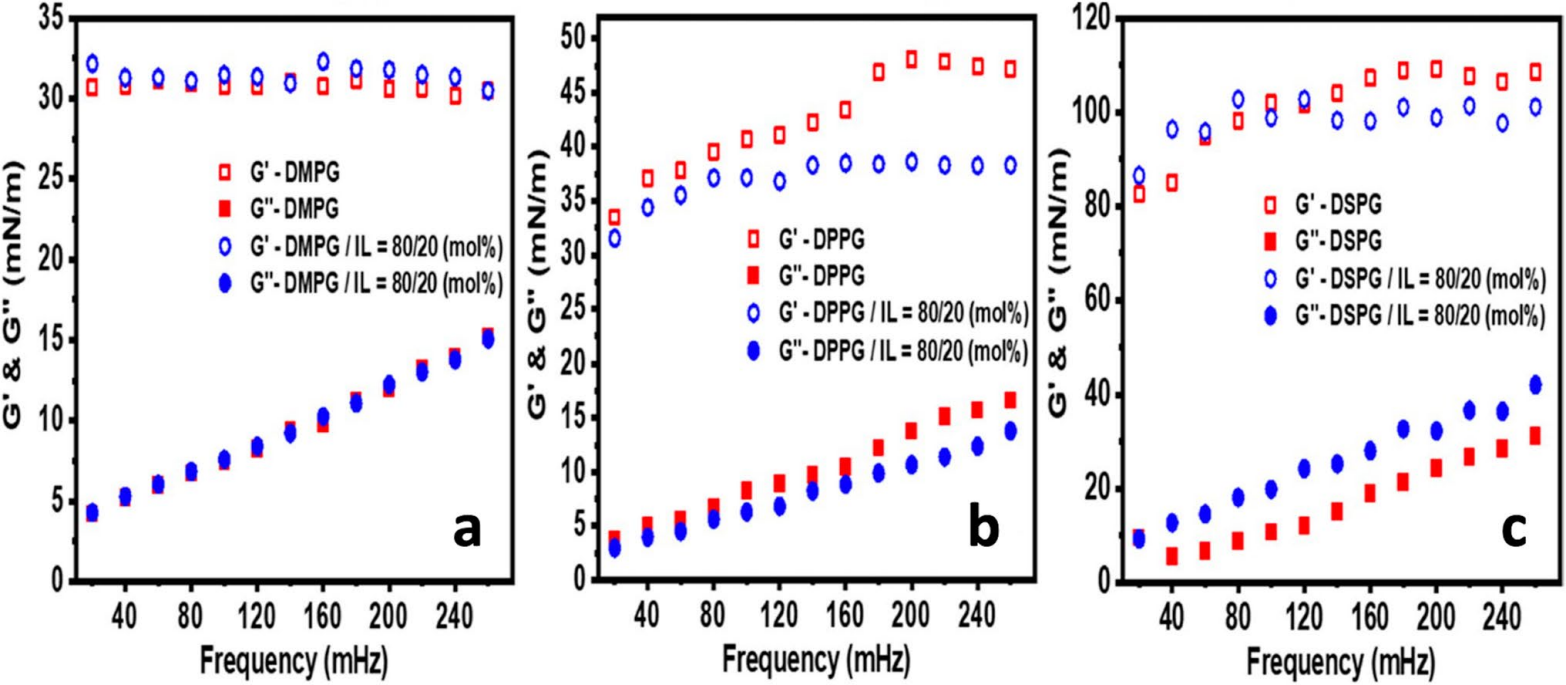
The effect of IL on the viscoelasticity of lipid monolayers from (Hitaishi et al. 2022). Frequency dependent dilation storage modulus (G′) and loss modulus (G″) for **a** DMPG, **b** DPPG, and **c** DSPG in the absence and presence of 20 mol % of [bmim][BF_4_]. Measurements were performed at 25° C at a surface pressure of 4mN/m where the lipid films are in the liquid extended phase. Figure reproduced with permission from the publisher

Ghosh and co-workers looked also at the effect of imidazolium-based ionic liquids on bilayers of anionic lipids like 1,2-dimyristoyl-sn-glycero-3-phosphoglycerol (DMPG) (Hitaishi et al. 2022). It has been nicely shown the important role of the thermotropic phase of the lipid in the lipid-IL interaction. For example, it resulted that the presence of imidazolium-based ILs in the lipid phase of DMPG makes the bilayer more ordered, while in the gel phase, by reducing the entropy, but disorders it while in the fluid phase. In the same work, the in-plane viscoelasticity has been measured by dilation rheology, oscillating the barriers of a Langmuir–Blodgett trough apparatus, and the effect of the ILs was recorded. For example, in the lipid liquid extended phase, it has been shown that both the storage and loss moduli of DPPG monolayers are reduced in the presence of [bmim][BF_4_] that, on the contrary, increases the loss modulus of DSPG monolayers, showing a role of the lipid chain length (Fig. 16). More recently, the role of cholesterol has been also investigated. Ghosh and co-workers looked at the effect of the imidazolium IL [dmim][Cl] on monolayers and multilayers of egg sphingomyelin lipid in the absence and presence of cholesterol (Hitaishi et al. 2023). Overall, it turned out that the presence of cholesterol in the lipid phase reduces the effects of the IL. For example, by means of X-ray reflectivity, it has been shown that in the absence of cholesterol the addition of the IL triggers the formation of interdigitated IL-rich domains that are barely forming in the presence of cholesterol.

## Mithu & Scheidt

**Fig. 17:**
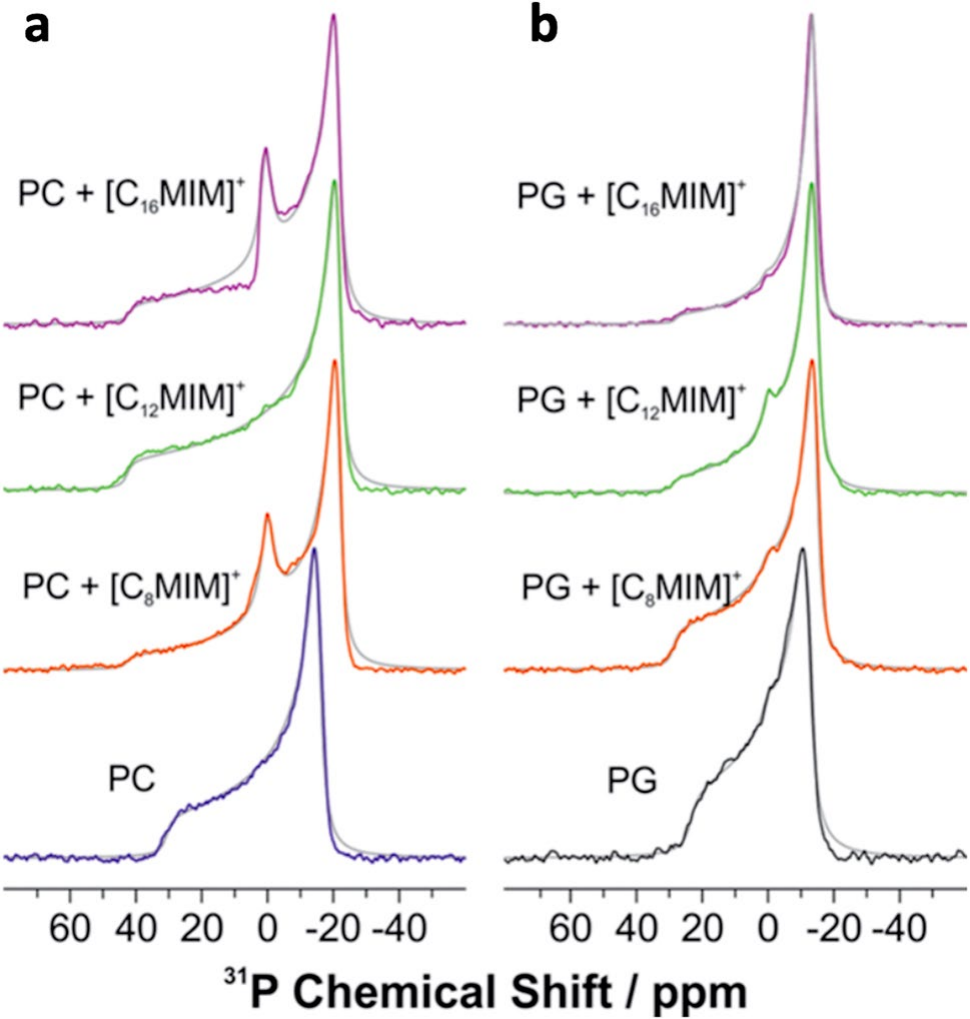
^31^P NMR spectra taken from (Kumar et al. 2019) of **a** POPC and **b** POPG in the absence and presence of [C_n_mim][Br] at a molar ratio of 0.768:1 at 25° C. Gray lines represent best fit numerical simulations of the ^31^P NMR lineshape. Reproduced with permission from the publisher

**Fig. 18:**
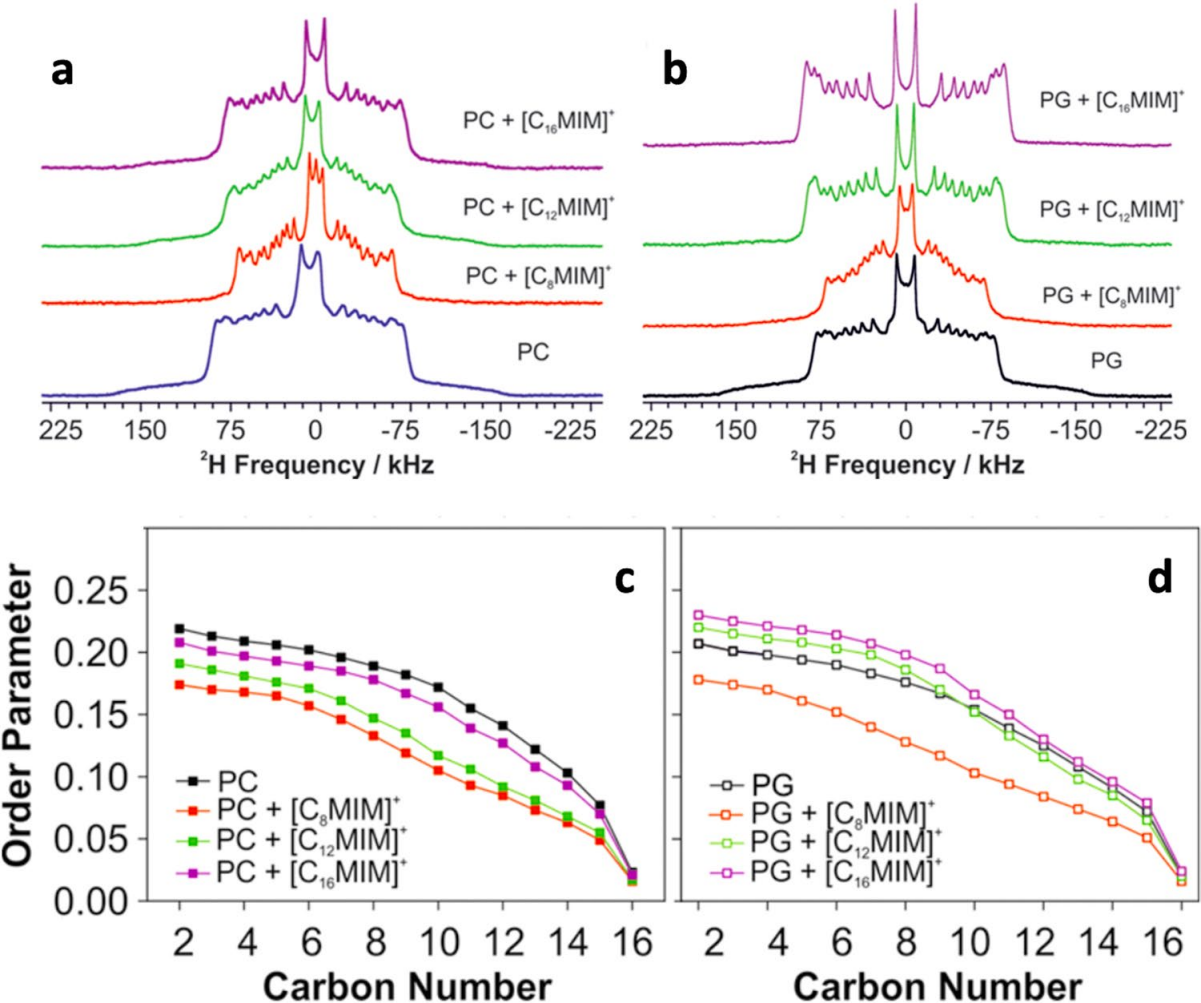
^2^H NMR spectra taken from (Kumar et al. 2019) of **a** POPC-d_31_ and **b** POPG-d_31_ multilamellar vesicles in the absence and presence of 0.768 mol of [C_n_mim][Br] per mole of lipid at 25° C, along with the extracted chain order parameter of the sn-1 chain of **c** POPC-d_31_ and **d** POPG-d_31_ membranes. Carbon number 16 corresponds to the methyl carbon in the palmitoyl lipid chain in the sn-1 position. Reproduced with permission from the publisher

Mithu and co-workers have contributed with a number of studies to the field. Their major approaches include solution-state NMR spectroscopy, DLS, zeta potential measurements, fluorescence-based dye-leakage assays and DSC. In one of their first work (Kumar et al. 2018) — to be best of my knowledge their first contribution to the field — they have looked at the interaction of the [C_12_mim][Br] IL with zwitterionic POPC membranes and anionic palmitoyl-oleoyl-phosphatidylglycerol (POPG) membranes. They have shown that the IL-cation inserts into the lipid membranes, driven primarily by the hydrophobic effect, and reaches with its imidazole ring the lipid-water interface of the lipid membrane near the upper chain/glycerol moieties. The presence of the IL-cation in the lipid region induces a strong decrease in the lipid order along the entire hydrocarbon chain for POPC; in contrast, in the case of POPG membrane, the upper half of the chain resulted more ordered while the lower half more disordered. The presence of the IL-cation makes the lipid membrane softer. Moreover, POPC membranes resulted more leakage-prone, but the IL-cation binding was observed to be stronger with POPG. In a follow-up work (Kumar et al. 2019), Mithu and co-workers looked also at the effect of IL chain length of the [C_n_mim]-cation with *n* = 8,12,16. They have shown that membrane affinity and permeability both correlate positively with IL chain length. Maximal dye leakage, for example, was observed for the [C_16_mim]-cation, while the [C_8_mim]-cation only induces a negligible leakage. The location of the IL-cation in the lipid membrane resulted to not be influenced by the IL chain length as well as the effect on the lipid head orientation and dynamics (Fig. 17). Concerning the lipid chains, all the [C_n_mim]-cations induce a decrease in the lipid chain order parameter in POPC membranes, with a maximum reduction observed for the shorter-tail IL-cation [C_8_mim]^+^. In contrast, the addition of [C_12_mim]^+^ and [C_16_mim]^+^ increases the order parameter of the lipid chains in POPG membranes (Fig. 18). The effect of these IL-cations on the membrane elasticity was also investigated. For POPC membranes, all the IL-cations make the membrane softer, with the stronger effect observed with the shorter-chain IL-cation [C_8_mim]^+^. In the case of POPG membranes, both [C_12_mim]^+^ and [C_16_mim]^+^ cations enhance membrane rigidity.

**Fig. 19:**
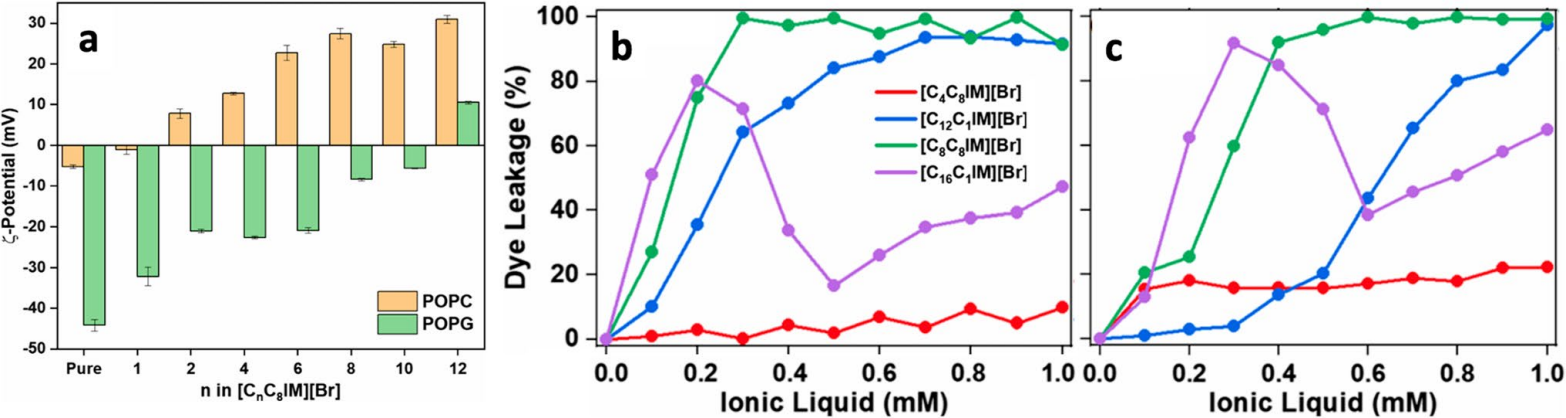
**a** Zeta Potential of POPC and POPG large unilamellar vesicles in the absence and presence of 1 mM [C_n_C_8_IM][Br] ionic liquids at 25° C. Dye leakage profiles of **b** POPC and **c** POPG large unilamellar vesicles after 10 min of addition of different concentrations of the ILs at 25° C. Taken from (Kaur et al. 2021a) and reproduced with permission from the publisher

Mithu and co-workers looked also at the effect of IL concentration spanning an extended range going from very low concentrations to above the CMC values (Kumar et al. 2020). They have shown that, as the IL concentration approaches the CMC value, lipid vesicles’ fusion occurs, resulting in unexpected quenching accompanied by a rapid dye leakage due to the formation of short-lived fusion-holes. Mithu and co-workers investigated also the effect of double-tail imidazolium-based ILs and compared it to the effect of the more traditional single-tail imidazolium-based IL carrying an equal number of carbon atoms but in the single alkyl chain (Kaur et al 2021a). Lipid membrane partitioning of the ILs was explored by means of zeta potential measurements, and membrane permeability by means of fluorescence-based dye-leakage assays (Fig. 19). They have shown that the membrane permeability increases following a sigmoidal trend with increasing the IL chain length, confirming the trend observed for single-chain ILs, even though these latter resulted in higher membrane permeabilization. Mithu and co-workers focused then on the role played by the IL-cation headgroup for which they employed solid-state NMR spectroscopy (Kaur et al. 2021b, 2022a). For these studies they used zwitterionic POPC lipid membranes (Kaur et al. 2021b) and POPG lipid membranes (Kaur et al. 2022a), and considered a set of different ILs, all carrying a dodecyl alkyl chain, based on imidazolium, pyrrolidinium, ammonium and few more. Overall, the lipid membrane permeability shows a strong dependence on the IL headgroup. Cations carrying an unshielded charge like imidazolium and ammonium induce strong reorientation of the POPC lipid headgroup, while large size cations like piperidinium in enhance POPC lipid chain dynamics.

In 2022, Mithu and co-workers published a work on the effect of the imidazolium IL [C_12_mim][Br] on permeability, structure, and dynamics of 2-lipids membranes made of POPC and POPG (Kumar et al. 2022). Membranes prepared at different molar ratios of the two lipids have been considered, and the focus was on the role of the lipid ratio. They have shown that, despite the highest membrane affinity, POPG-rich membranes are less permeable. The contrary has recorded in POPC-rich membranes. Interestingly, it was observed that the variation of surface charge or membrane affinity does not correlate with the amount of negatively charged POPG in the membrane. Solid-state NMR revealed that the minor POPC component in POPG-rich membranes is homogeneously dispersed, while the minor POPG component in POPC-rich membranes is phase-separated. Mithu and co-workers looked also at the effect of di-cationic ionic liquids (Kaur et al. 2022b). In this contest, by solid-state NMR spectroscopy, they investigated the interaction mechanism of two of these ILs with POPC and POPG lipid membranes. It turned out that di-cations with a short linker and long terminal chains cause substantial perturbation to the bilayer structure, even higher than the corresponding mono-cations, despite the low toxicity and membrane permeability. The last contribution to the field by Mithu and co-workers focused on the role of cholesterol. They looked at the interaction between the imidazolium-based IL [C_12_mim][Br] and cholesterol-containing POPC and POPG membranes, showing that the presence of cholesterol triggers a reduction in membrane permeability (Kumar et al. 2023).

## Yu

**Fig. 20:**
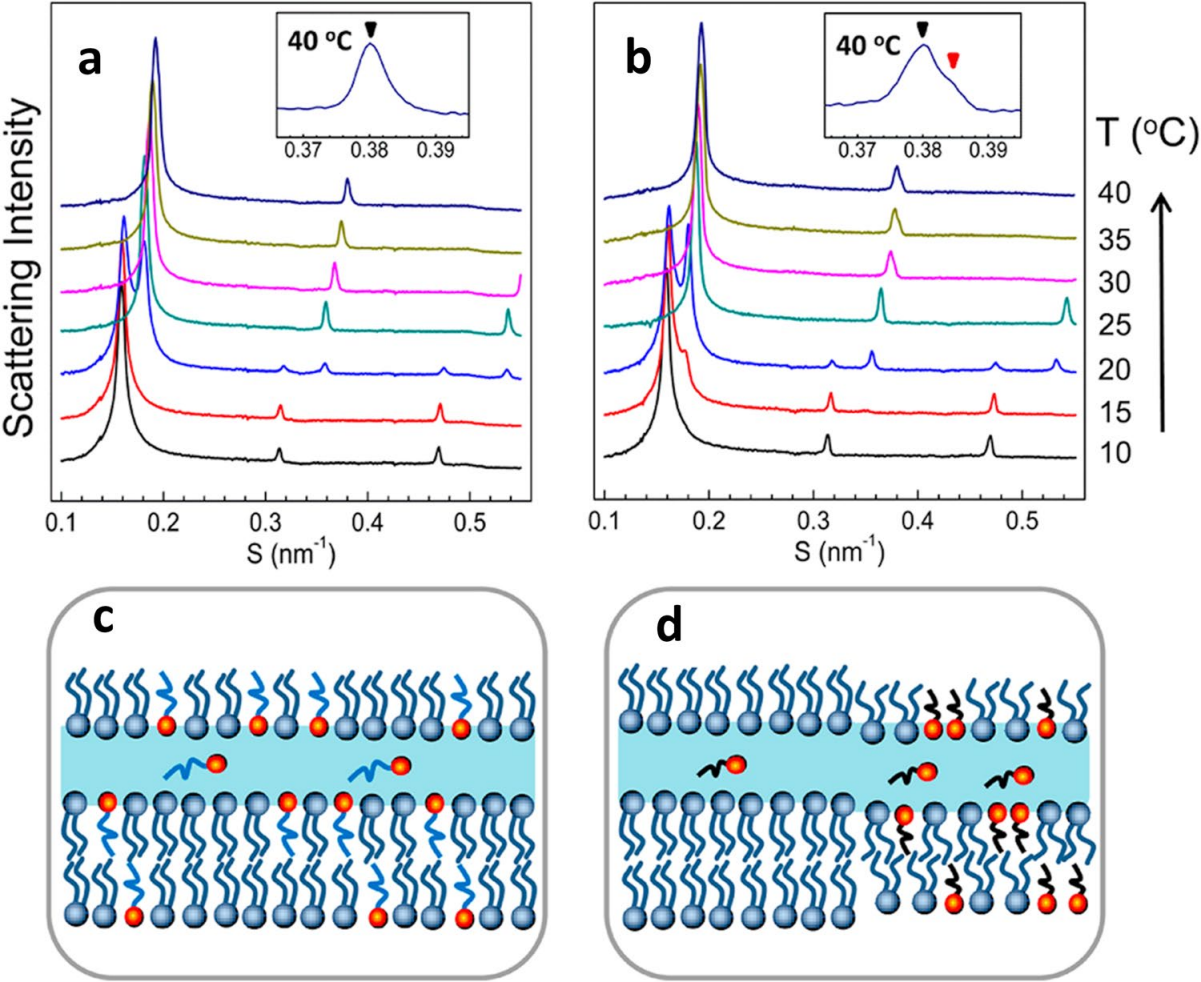
SAXS data of aqueous dispersions taken from (Guo et al. 2019) of **a** [C_16_mim][Cl]-POPE mixture and **b** [C_12_mim][Cl]-POPE mixture during heating scans at IL:POPE molar ratio of 1:35. The insets show the magnified second-order diffractions at 40° C. **c** and **d** report schematic illustrations of the membrane structures of the two mixtures, showing the more destabilizing effect played by the shorter-tail IL. Figure reproduced with permission from the publisher

Yu and co-workers have contributed with a couple of studies to the field. Their major approaches includes DSC, and synchrotron-based SAXS and wide-angle X-ray scattering (WAXS), usually performed at different temperature covering the lipid phase transitions. In one of their first work (Guo et al. 2019) — to be best of my knowledge their first contribution to the field — they have investigated the effect of a set of imidazolium-based ILs, differing in the length of their hydrocarbon chain, on 1-palmitoyl-2-oleoyl-sn-glycero-3-phosphoethanolamine (POPE) lipid assemblies (i.e., vesicles an aqueous dispersions). SAXS data highlighted the role played by the IL hydrocarbon tail length in respect to the lipid hydrocarbon tail length. It resulted that, when the IL and lipid tail lengths are matching, the destabilizing effect of the IL decreases strongly (Fig. 20).

**Fig. 21:**
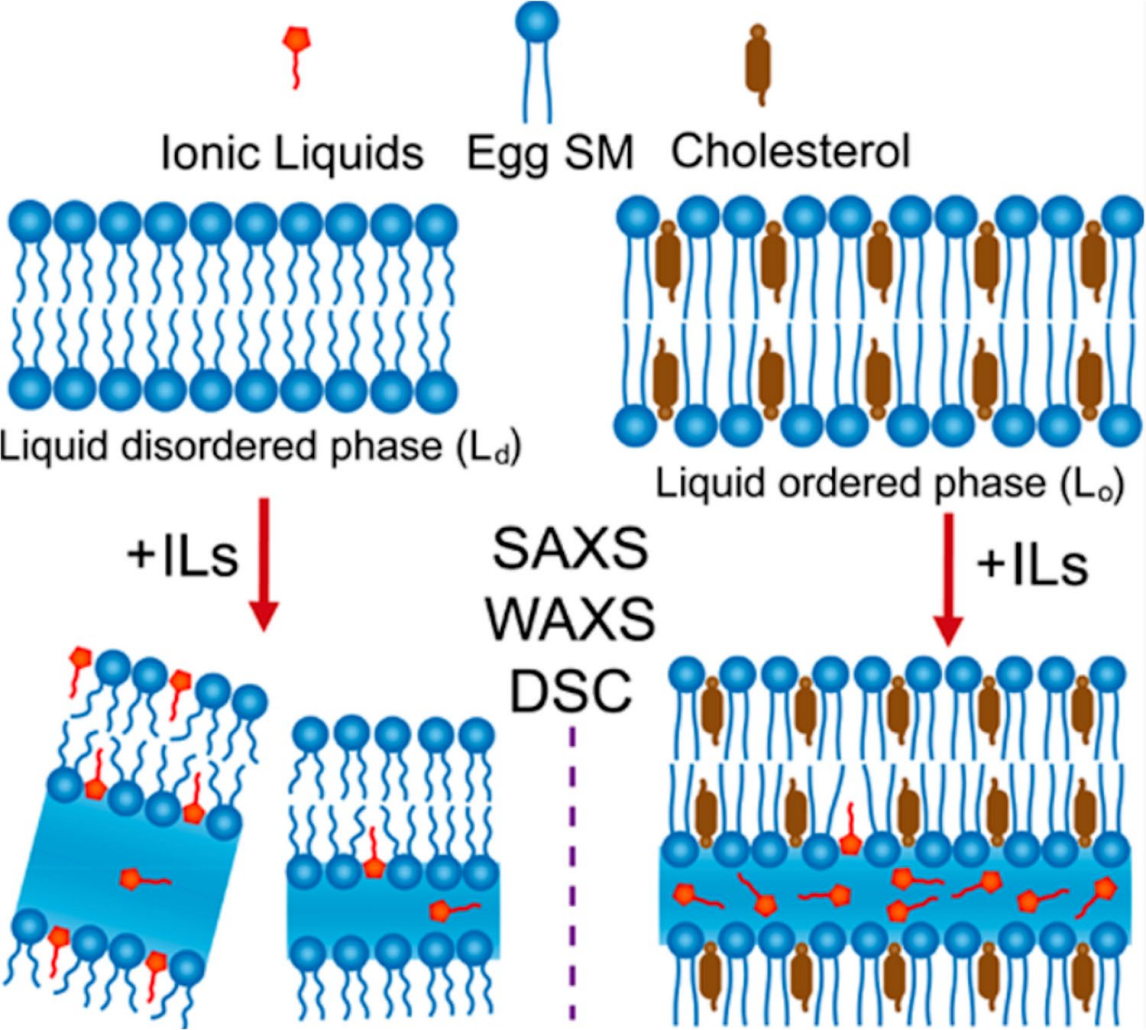
A schematic representation of the role of cholesterol helping the lipid bilayer against the destabilizing effect of ILs taken from (Hao et al. 2022). Figure reproduced with permission from the publisher

Yu and co-workers investigated also the effect of two imidazolium-based ILs on DPPC lipid systems up to 1:5 lipid:IL molar ratio (Hao et al. 2021). DSC profiles shown that the longer-tail IL ([C_6_mim][OAc]) reduces by about 5 degrees the phase transition temperature of the lipid system, but no measurable effect was observed for the shorter-tail IL ([C_4_mim][OAc]). SAXS data suggested that, in the gel lipid phase, the longer-tail IL inserts into the lipid region and induces interdigitation of the lipids, while the shorter-tail IL is not able to diffuse into the lipid region. By means of SAXS, WAXS, and DSC, Yu and co-workers have then looked at the role of cholesterol in the interaction between imidazolium-based ILs with egg sphingomyelin lipid membranes (Hao et al. 2022). They found that, in the absence of cholesterol, even at the very low lipid:IL molar ratio of 1:0.01, the ILs affect the integrity of the lipid bilayer structure. However, the presence of cholesterol in the lipid region helps the membrane to resist against the damaging effect of the ILs (Fig. 21).

## Podestà

**Fig. 22:**
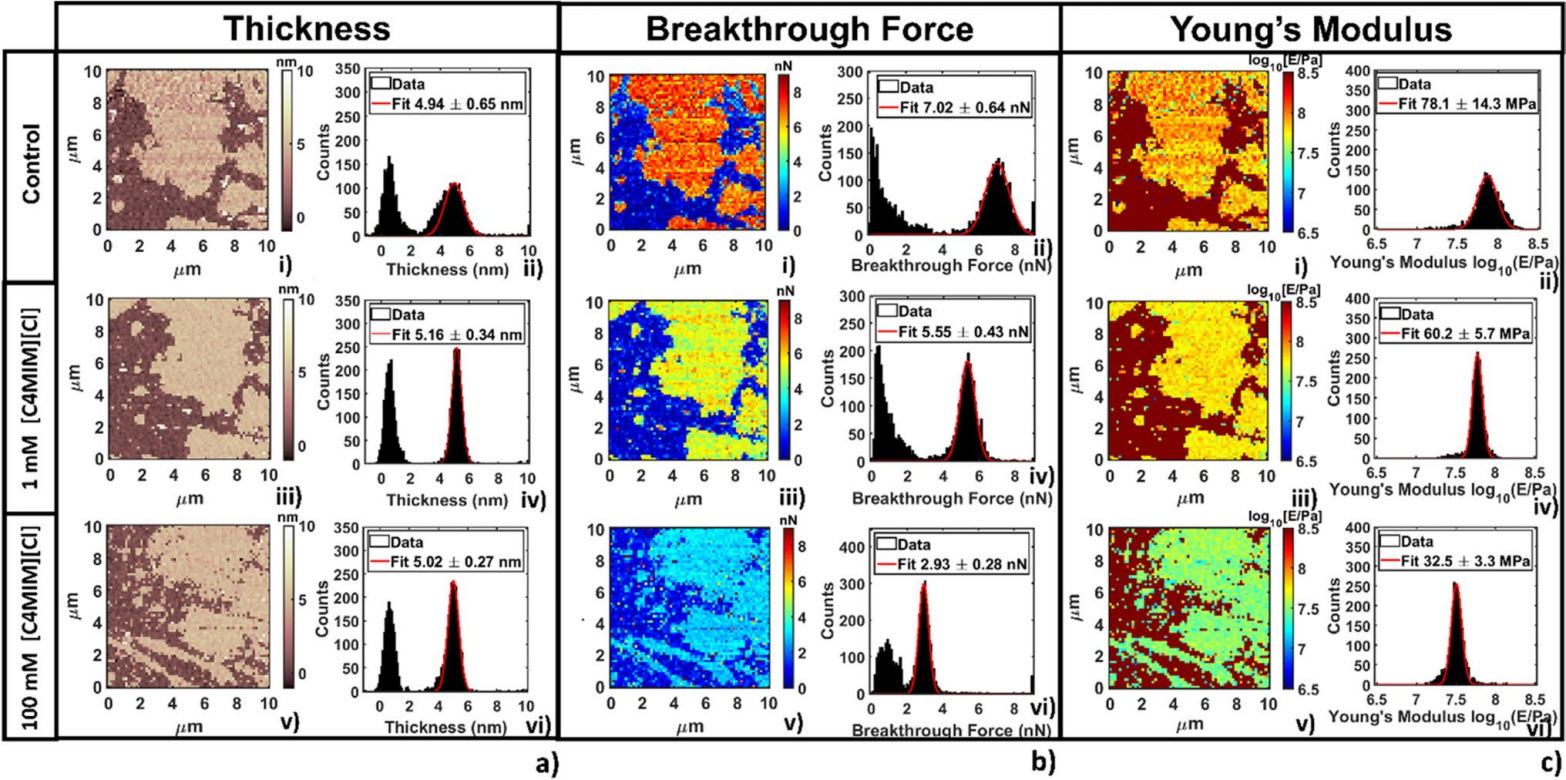
Representative analysis of the evolution of **a** thickness, **b** breakthrough force, and **c** Young’s modulus for DOPC supported bilayer varying the concentration of [C_4_mim][Cl] taken from (Galluzzi et al. 2022). For each panel, image (i) represents the map, and image (ii) the associated histogram with Gaussian fit. In the same order (iii, iv, and v, vi) images represent the same location after 20 min of interaction with 1 mM and 100 mM concentration of the IL, respectively. Figure reproduced with permission from the publisher

Podestà and co-workers contributed to the field with two studies (Galluzzi et al. 2013, 2022). They looked at the effect of a number of imidazolium-based ILs, of different hydrocarbon chain length and hydrophilicity/hydrophobicity, on the structural integrity, morphological, mechanical, and electrostatic properties of solid-supported DOPC lipid monolayers and bilayers, as a function of IL-concentration and IL incubation time. Overall, it turned out that the ILs trigger the formation of lipid-IL complexes/domains resulting in an overall decrease of Young’s modulus, area stretching modulus and breakthrough force, which magnitude has shown to depend on the IL concentration at the bilayer/water interface (Fig. 22). Podestà and co-workers contributed also with an AFM study focused on the effect of imidazolium ILs on the morphology and rigidity of the model cell line MDA-MB-231 (Galluzzi et al. 2018). They have shown that ILs induce modifications of both cell morphology and overall cell rigidity by acting on the physical properties of the outer cell layer, linked to the actin cytoskeleton, with stronger effects recorded for longer-chain ILs and at higher IL concentrations.

## Vaden

**Fig. 23:**
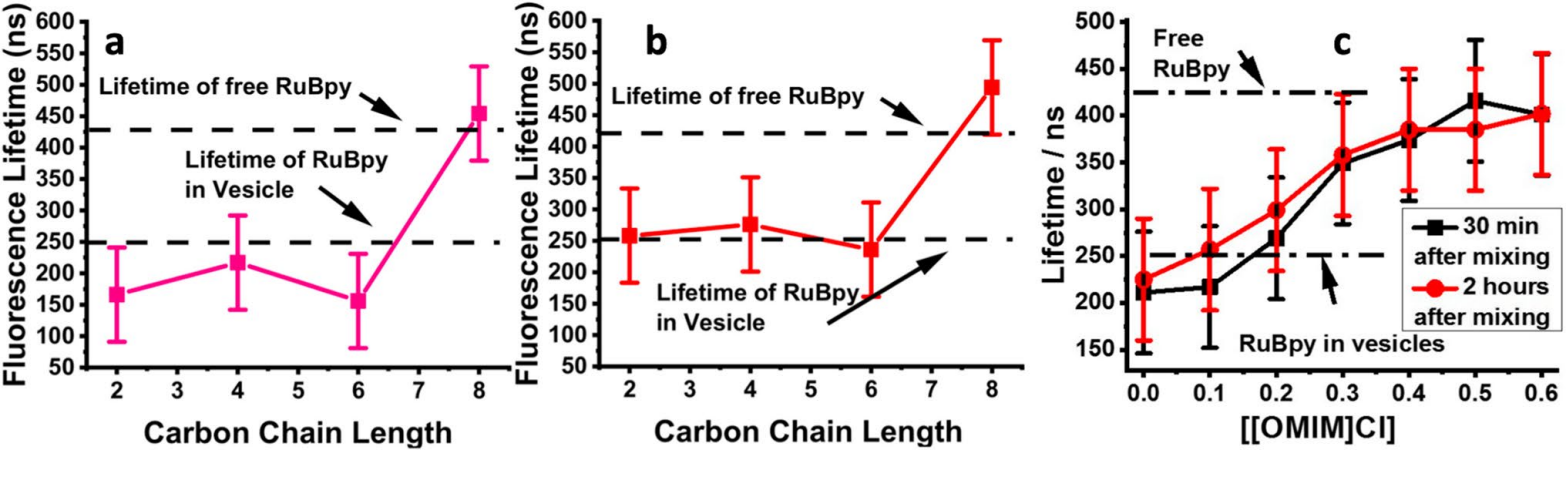
Laser-induced fluorescence lifetimes of **a** DOPC:DOPG (98:2) and **b** DOPC:DOPG:Cholesterol (70:2:28) lipid unilamellar vesicles entrapped with Ru(bpy)_3_^2+^ in the presence of 0.5 M [C_n_mim][Cl] for IL-chain lengths of *n* = 2,4,6,8. **c** Laser-induced fluorescence lifetimes of DOPC:DOPG (98:2) lipid unilamellar vesicles entrapped with Ru(bpy)_3_^2+^ as function of [C_8_mim][Cl] concentration. Taken from (Cook et al. 2019) and reproduced with permission from the publisher

Vaden and co-workers contributed, to the best of my knowledge, with two works to the field (Hanna et al. 2017; Cook et al. 2019). By means of trapped-fluorophore fluorescence lifetime-based leakage experiments and DLS, they assessed the effect of a set of imidazolium ILs on the stability and permeability of DOPC:DOPG (98:2 molar ratio) and DOPC:DOPG:cholesterol (70:2:28 molar ratio) lipid membranes. They have shown that the longer-tail IL 1-methyl-3-octylimidazolium chloride ([omim][Cl]) is able to permeabilize the lipid vesicles at an IL-concentration corresponding to its CMC (Fig. 23).

## Wiedmer

**Fig. 24:**
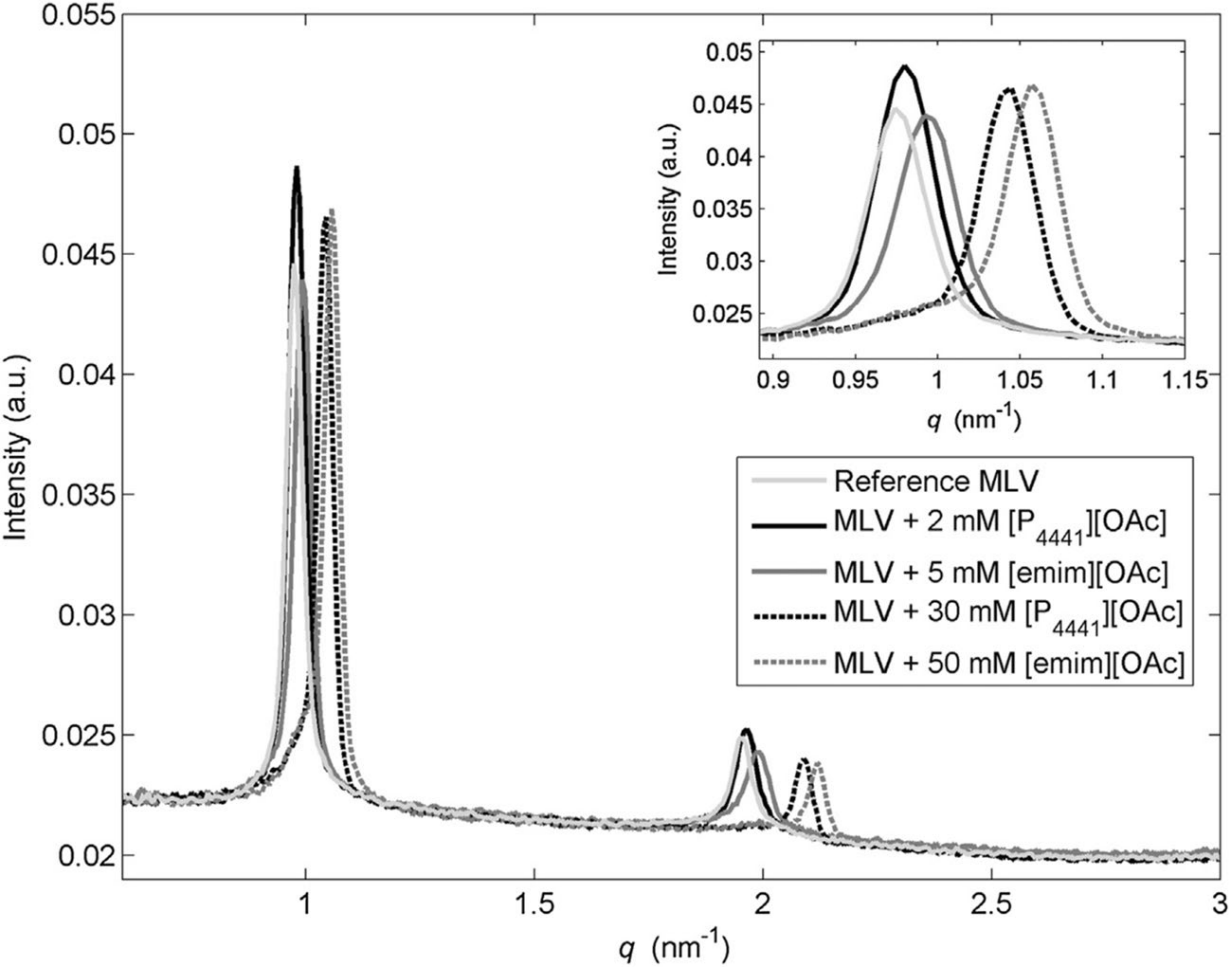
The effect of [P_4441_][OAc] and [emim][OAc] ILs on the SAXS pattern of POPC multilamellar vesicles (MLV). Neat MLV (light gray), MLV treated with [P_4441_][OAc] (black), and MLV treated with [emim][OAc] (dark gray). Insert: magnification of the first diffraction peak. Taken from (Kontro et al. 2016) and reproduced with permission from the publisher

Wiedmer and co-workers have contributed with several studies to the field. Their major approaches include SAXS, nanoplasmonic sensing, QCM, DLS, zeta potential measurements, and DSC. In one of their first work (Duša et al. 2015) — to be best of my knowledge their first contribution to the field — they investigated the effect of two phosphonium-based ILs on negatively charged unilamellar eggPC:POPG liposomes They showed that the longer-chain phosphonium IL [P_14444_][Cl] associates more strongly with the liposomes compared to the shorter-chain phosphonium IL [P_8444_][Cl], and both ILs change the viscoelasticity of the liposomes at moderate IL concentrations. At higher IL concentrations (partial) vesicle rupture occurs, and above the CMC values the dissolution of the vesicles eventually happens. The effect of a set of amidinium-, imidazolium-, and phosphonium-based ILs on the size and surface charge of liposomes (eggPC:POPG 75:25 and eggPC:POPG:cholesterol 50:25:25) as well as on their electrophoretic mobility was then studies by Wiedmer and co-workers (Mikkola et al. 2015). The more hydrophobic ILs were found to have a major effect on the surface charges and sizes distributions of the liposomes. In a follow-up work (Kontro et al. 2016), they have looked at the effect of phosphonium-based ionic liquids on lipid membranes of POPC, eggPC:eggPG (80:20 mol%) and eggPC:eggPG:cholesterol (60:20:20 mol%). They have shown that the addition of the ILs induces a decrease of the liposomes’ lamellar spacing (Fig. 24) or the complete disruption of the liposomes, with the magnitude of the effect positively correlated with the IL concentration. In the case of unilamellar vesicles, upon addition of the ILs, a decrease in vesicles’ size was recorded first followed, at higher IL concentrations, by the aggregation of vesicles. Inclusion of cholesterol resulted in lowering the destabilizing effect of the IL, e.g., the [P_8881_][OAc] IL ruptures lipid vesicles without cholesterol at lower concentrations than vesicles with cholesterol. At higher IL concentrations, it was observed the reassembling of the rupture liposomes into organized lamellae.

**Fig. 25:**
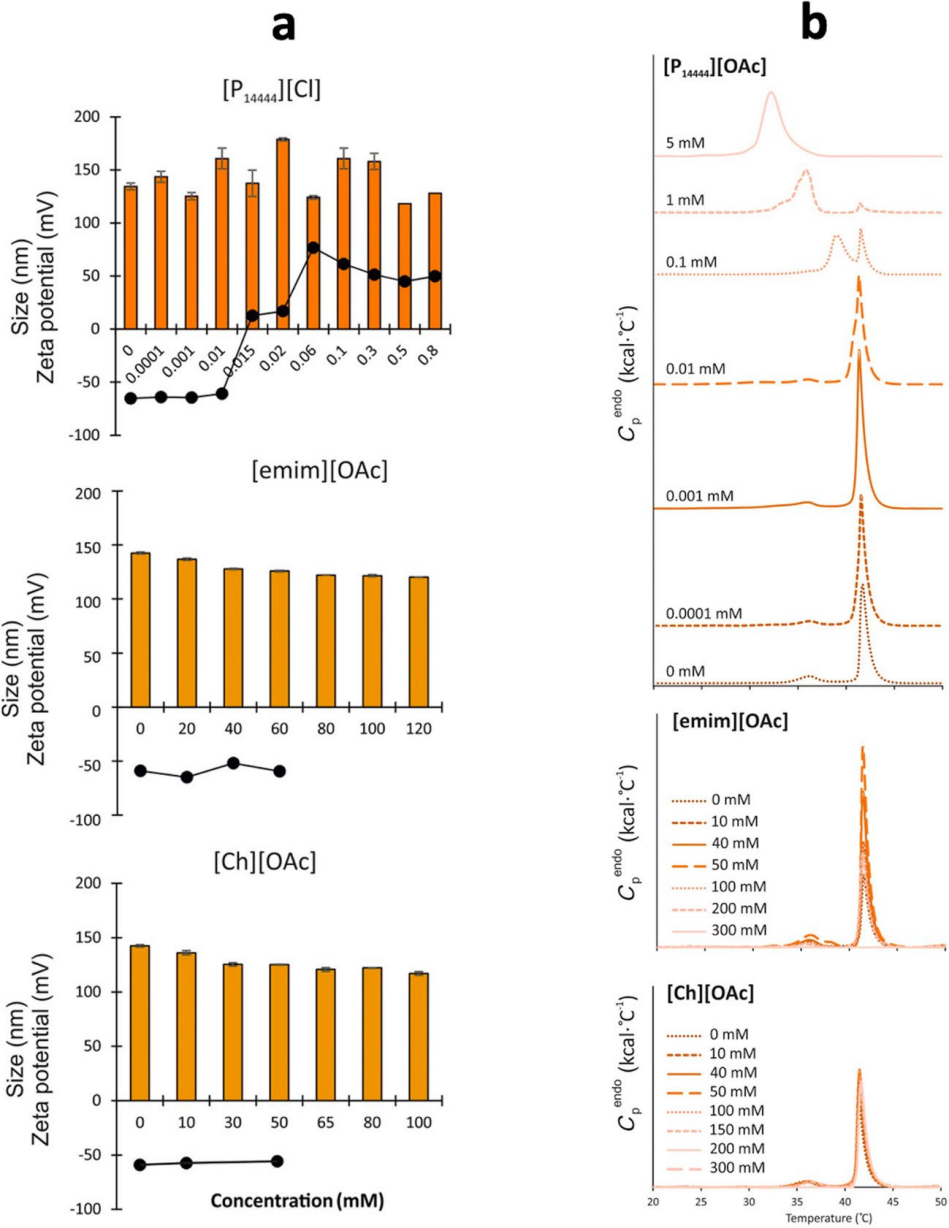
**a** Effect of ILs on size and zeta potential of eggPC:eggPG (80:20 mol%) liposomes. **b** Effect of ILs on the phase transition temperature of DPPC liposomes. Taken from (Ruokonen et al. 2018) and reproduced with permission from the publisher

In a follow-up work (Witos et al. 2017), Wiedmer and co-workers used nanoplasmonic sensing to look at the effect of amidinium- and phosphonium-based ILs on substrate-immobilized POPC lipid vesicles and single lipid bilayers. Depending on the IL, real-time binding and/or lipid removal were observed. The strongest effect was observed with [P_14444_][OAc] that caused the deformation of the vesicles and consequent lipid removal, while for [P_4441_][OAc] simple binding and a more superficial interaction was recorded. Wiedmer and co-workers investigated then the role of cholesterol, by means of DSC and nanoplasmonic sensing (Russo et al. 2017). Overall, the presence of cholesterol inhibited/delayed the IL binding and infiltration into the lipid membrane as well as the lipid depletion. By DLS, zeta potential measurements, DSC, and NMR spectroscopy, Wiedmer and co-workers investigated the effect of different ILs, including several phosphonium-based ILs (Ruokonen et al. 2018). A variety of different interactions were observed. For example, some ILs do not alter the liposome’s zeta potential, e.g., [emim][OAc], whereas other ILs can even revert it, e.g., [P_14444_][Cl] (Fig. 25a). Interestingly, DSC data reproduced a similar trend, showing that the ILs able to alter the liposome’s zeta potential are also able to strongly affect/reduce the main phase transition temperature of the lipid, in a IL concentration-dependent manner (Fig. 25b). In the same work, NMR spectroscopy was employed to measure the diffusion coefficients of the ILs while alone (in bulk) or in the liposomes’ solution, showing, for example, the ability of ILs to form aggregates in the bulk (a CMC determination method), and to aggregate with the disrupted liposomes to form IL-lipid aggregates. In what is, for the best of my knowledge, their last contribution to the field so far (Rantamäki et al. 2019), Wiedmer and co-workers carried out a steady-state fluorescence anisotropy study looking at the interactions between (non-surface-active) ILs and liposomes. This technique, using a fluorescent probe, allows to probe the lipid bilayer local environment and access the bilayer micro-viscosity. Four liposome compositions were used, i.e.: POPC, and mixtures of POPC, POPS and cholesterol, with the POPC:POPS:Chol 55:20:25 mixture correlating very well with a set of toxicity studies. They have shown that, depending on the ILs, the bilayer micro-viscosity can either increase or decrease, with the magnitude of the variation depending on the lipid composition. For example, the phosphonium IL [P8881][OAc], considered the most destructive IL tested in the study, decreases by about 20% the micro-viscosity of the POPC bilayer and by about 40% the micro-viscosity of the POPC:Chol (75:25 molar ratio) mixed bilayer, but anyway maintained above the 100 mPa⋅s threshold typical for biological membranes. A similar trend was observed for the change/decrease in anisotropy induced by the ILs, for which a decrease designates an increase in rotational freedom of the fluorescent probe, usually due to increased disorder of the lipid chains.

## Maginn

**Fig. 26:**
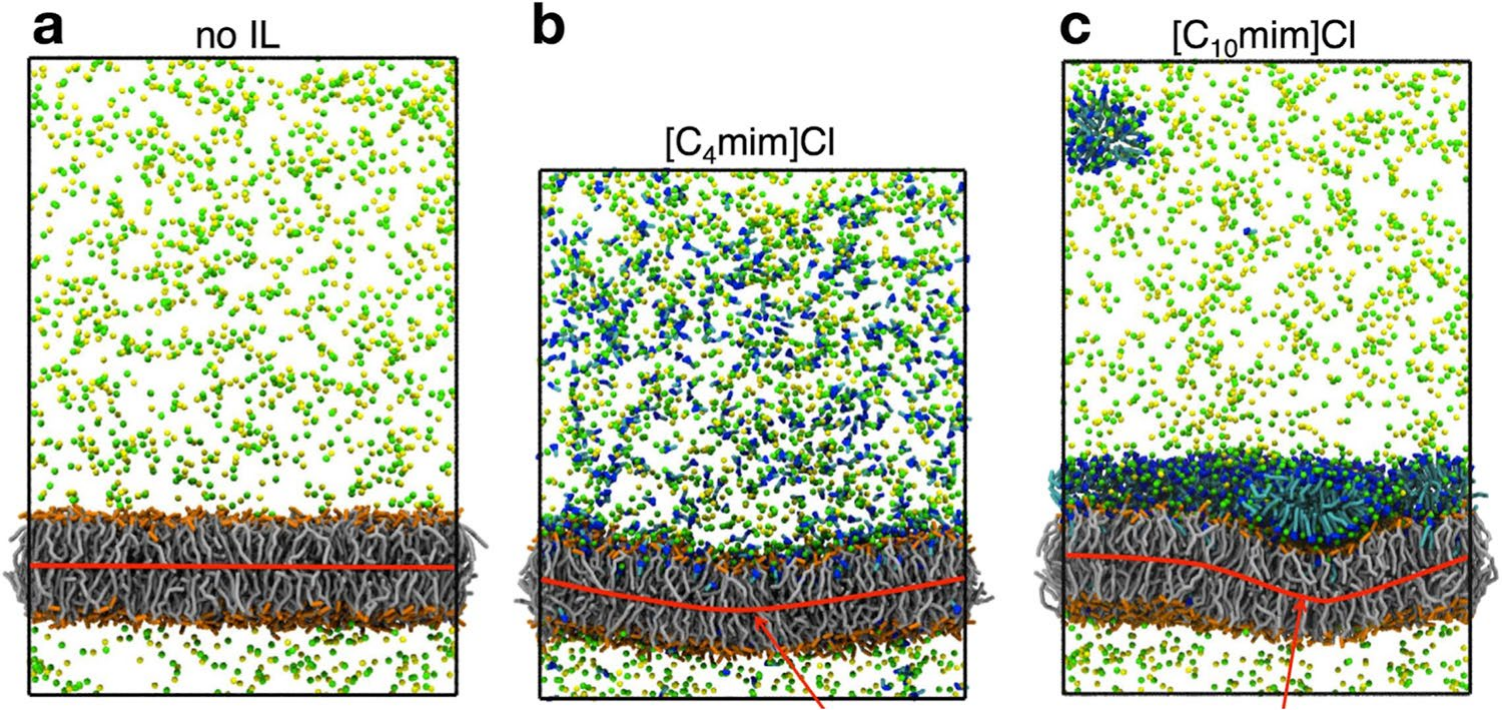
Snapshots of coarse grained simulations of a POPC bilayer system **a** without ILs, **b** with [C_4_mim][Cl], and **c** with [C_10_mim][Cl] in NaCl buffer (top row). Coarse grained water molecules are not displayed. Interactions between IL-cations with the POPC bilayer induce the bending of the bilayer. In **c** a nearly fully covered monolayer of absorbed [C_10_mim][Cl] is formed at the bilayer/aqueous interface. Taken from (Yoo et al. 2016a) and reproduced with permission from the publisher

Maginn and co-workers have contributed with several studies to the field. Their major approaches are MD computer simulations (both full-atom and coarse-grained), which they combine with several other (experimental) approaches. In one of their first work (Yoo et al. 2014) —to be best of my knowledge their first contribution to the field — they have looked at the interaction of imidazolium-based ILs with a POPC lipid bilayer. IL-cations of different chain length ([C_n_mim] with n = 4,8,12) paired with different counterions ([Cl], [BF_4_], [PF_4_], [NTf_4_]) have been studied to assess the role of IL-chain length and IL-anion in the interaction. They have shown that the IL-cations spontaneously insert into the lipid bilayer, with some of the cation types also showing the ability to “flip” inside the lipid bilayer from the outer/initial leaflet to the inner/opposite leaflet, depending on their chain length. Several structural and dynamical properties have been computed from the MD trajectories, including area per lipid, lipid lateral diffusion coefficient, order parameters, and number of inserted IL-cations. In a follow-up work (Yoo et al. 2016a), Maginn and co-workers combined coarse-grained MD simulations with confocal laser scanning microscopy to look at the effect of [C_n_mim][Cl] ILs on a POPC lipid bilayer. The confocal measurements shown destabilizing effect of the ILs that induce the formation of multilayers, fibers, vesicles up to the complete disruption of the lipid bilayer, which occurs around the CMC of the ILs. The measurements also confirmed the absorption of the ILs into the lipid region. The results of the MD simulations supported the experimental results and provided a microscopic-level picture of the interaction. They have shown, for example, that the number of inserted [C_4_mim]^+^ cations into the upper bilayer leaflet reached a saturation limit of approximately 0.6 inserted cations per lipid and, in few nanoseconds after saturation, the bilayer undergo a long-wavelength bending in response to the asymmetric distribution of the inserted cations (Fig. 26). Consistently, the bending modulus dropped from approximately 22.6 × 10^−20^ J to 9.4 × 10^−20^ J, upon incubation in the IL. By means of coarse-grained MD simulations, Maginn and co-workers looked also at the effect of these imidazolium-based ILs on several different lipid bilayer morphologies, i.e., lipid bilayer, bilayer disk, double bilayer, and vesicles (Yoo et al. 2016b). They have shown that the asymmetry insertion of IL cations into one side of a lipid bilayer leaflet enhances the leaflet strain, which can lead to a morphological disruption in the bilayer. The asymmetric IL absorption causes a decrease of the bilayer bending modulus by one or two orders of magnitude relative to that of the IL-free bilayer.

## Evans – The kick-off contributions in the field

**Fig. 27:**
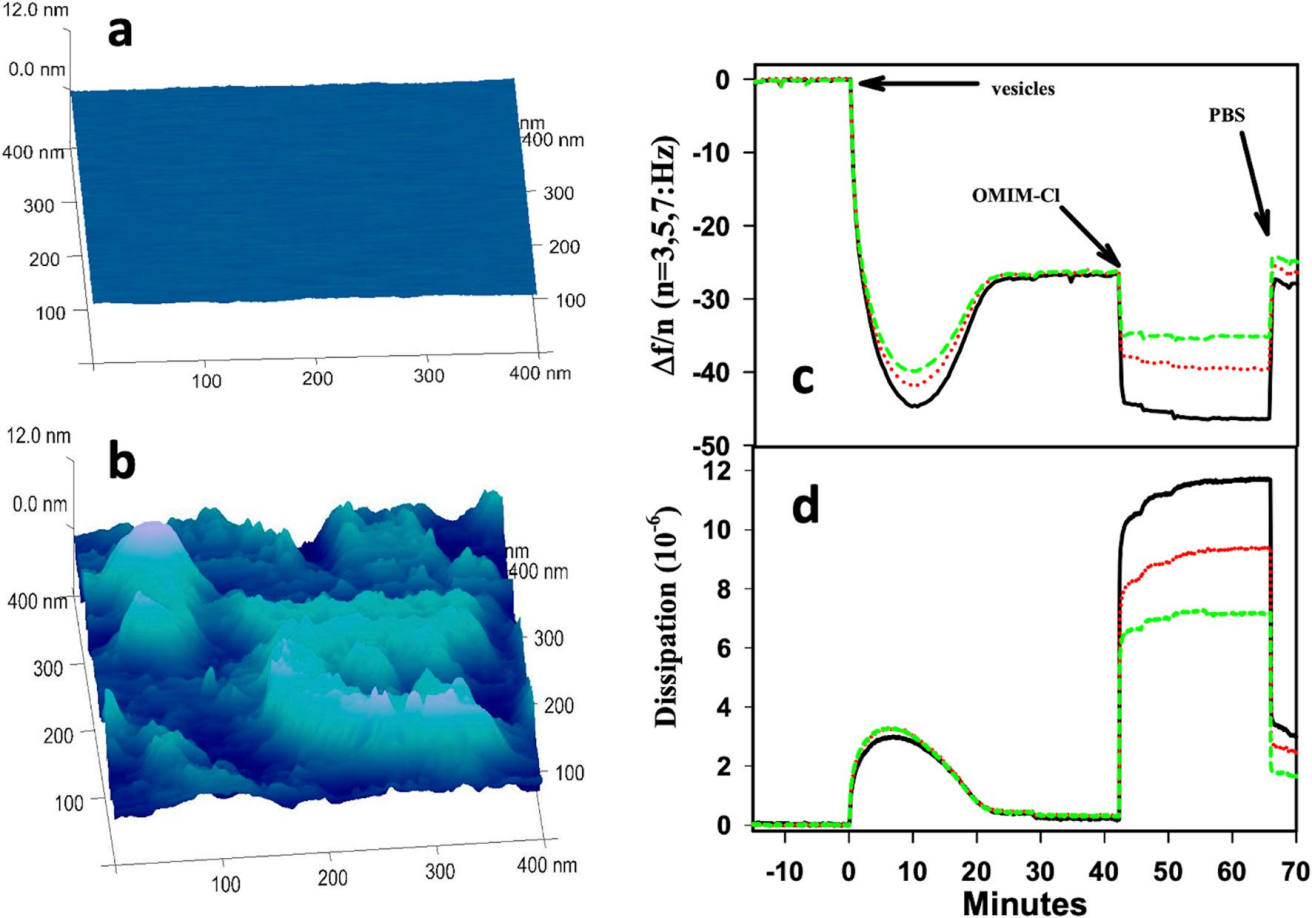
AFM image of DEPC lipid bilayer **a** prior and **b** after 15 min of exposure to [omim][Cl]. **c** Quartz crystal microbalance with **d** dissipation response at 15 (black), 25 (red), and 35 (green) MHz as DEPC vesicles form single supported lipid bilayers and interact with [Omim][Cl] at 20° C. Taken from (Evans 2008a) and reproduced with permission from the publisher

Evans has contributed with several studies to the field. His major approaches include QCM, AFM, DLS, and fluorescence leakage assays. In his first work (Evans 2006) — to be best of my knowledge the first contribution to the field ever — he has looked at the effect of imidazolium-based ILs on the structural integrity of DOtPC lipid large unilamellar vesicles, showing that these ILs induce leakage in lipid vesicles and shrink of the vesicles. He has shown that the longer the IL hydrocarbon chain, the higher the induced instability of the bilayers. Encapsulated material was released from the vesicles either through small holes in the bilayer or due to the full-vesicles disruption, depending on the IL in use and its concentration. In a follow-up work (Evans 2008a), Evans investigated the effect of a set of imidazolium and pyrrolidinium ILs on the morphology of supported lipid bilayers on silica. He has shown, for example, that [omim][Cl] removes lipids from the bilayer and increases its roughness (Fig. 27), while [BMP][Tf_2_N] forms a film over the supported bilayer. Evans looked also at the effect of [bmim][Cl] on DMPG:DEPC (2:98 molar ratio) lipid vesicles and single supported bilayers absorbed on gold, silica, and gold-modified solid surfaces (Evans 2008b). Depending on the surface, either lipid vesicles or single lipid bilayers attach/form on it, and then the effect of the IL was investigated by QCM. It turned out, for example, that [bmim][Cl], even above its CMC value, is not able to remove/disrupt the lipid vesicles attached on gold, but it is able to remove lipids from the single supported bilayer formed on the modified-gold surface. This study highlight the potentially-key role played by the substrate-lipid bilayer interaction in studying IL-lipid bilayer interaction on substrates.

## Kashyap

**Fig. 28:**
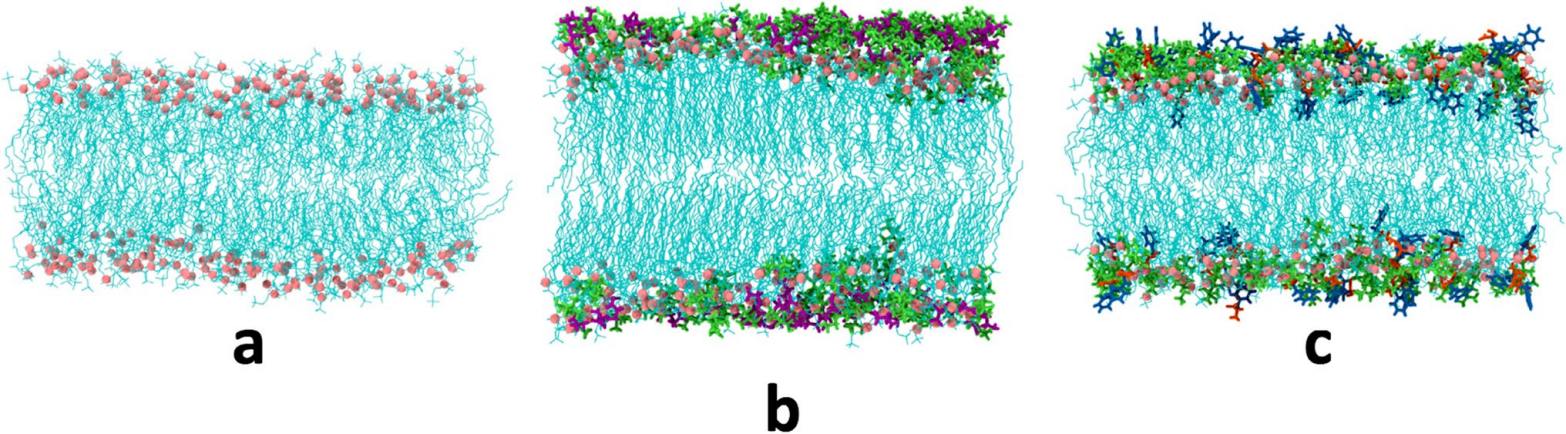
Snapshots representing DMPC lipid bilayer **a** in water, **b** in 20 mol % of [Ch][Gly], and **c** in 10 mol % [Ch][Phe]. Taken from (Shobhna et al. 2021) and reproduced with permission from the publisher

Kashyap and co-workers have contributed with a set of MD simulation studies to the field. In one of their first work (Kumari and Kashyap 2019) — to be best of my knowledge their first contribution to the field — they investigated the effect of the [Ch][Gly] IL on POPC and POPE lipid bilayer. They have shown that [Ch][Gly] affect more POPC bilayers than POPE bilayers, by shrinking them and order their hydrocarbon tails, with the [Ch]^+^ cation penetrating the bilayer. In a follow-up work (Kumari et al. 2020c), they extend their investigations looking at the effect on another cholinium-based IL, i.e., [Ch][Phe], showing that it destabilizes more POPE bilayers than POPC bilayers, with the [Phe] ^−^ anions diffusing into the hydrophobic region of the bilayer and inducing the accumulation of [Ch]^+^ cations at the bilayer-water interface. They looked then at the effect of these two cholinium-based ILs on DMPC lipid bilayers (Shobhna et al. 2021). It turned out that [Ch][Gly] affects the bilayer the most, inducing a phase transition from fluid to gel phase at 20% mol of the IL (Fig. 28). [Phe] ^−^ anions tends to diffuse into the bilayer, while [Gly] ^−^ anions accumulates mainly around the lipid headgroups.

## Warr

**Fig. 29:**
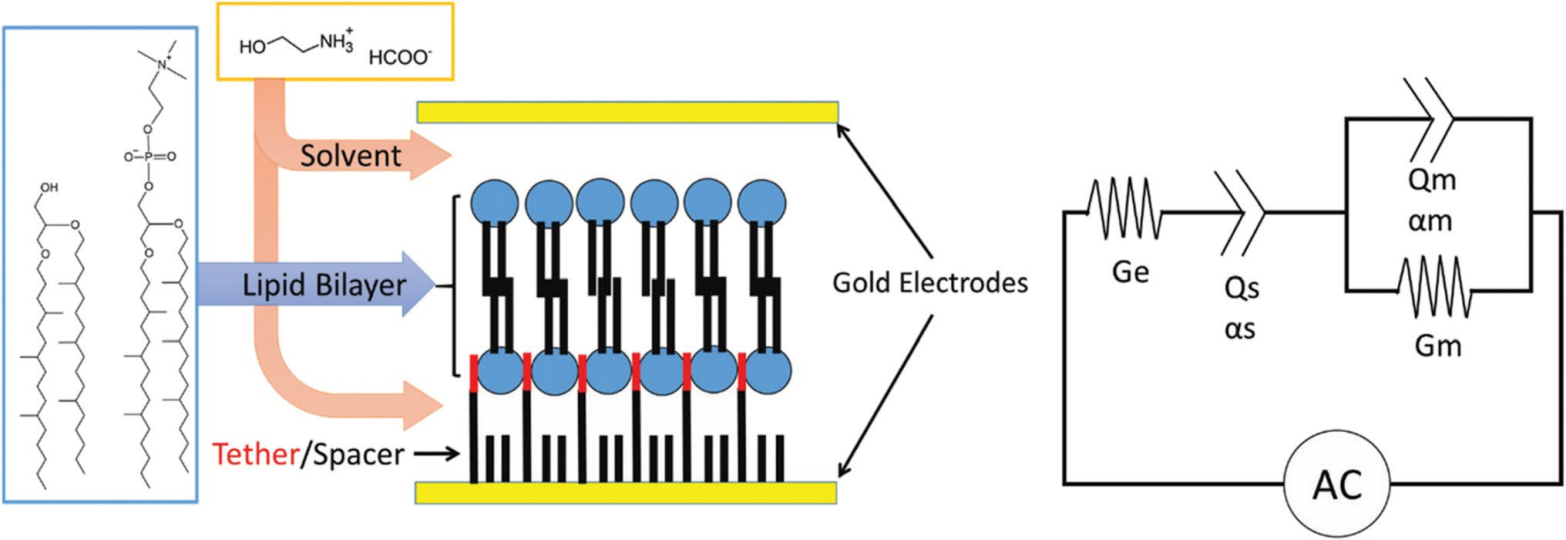
Schematic of the tethered bilayer AM199 comprised of diphytanyl-etherphosphatidylcholine and glyceroldiphytanylether lipids immersed in water of IL solvent tethered between gold electrodes alongside single circuit model used to analyze impedance spectra of tethered membranes. Ge — conductance of the electrolyte, Qs/αs — capacitance of the series and associated constant phase element, Qm/αm — membrane capacitance and associated constant phase element, and Gm — membrane conductance. Taken from (Bryant et al. 2021) and reproduced with permission from the publisher

Warr and co-workers have contributed with two studies to the field. In one of their first work (Bryant et al. 2021) — to be best of my knowledge their first contribution to the field — they have shown that a tethered lipid bilayer (AM199 lipid mixture) can be assembled directly in a pure IL environment (ethanolammonium formate), and retains its structure upon exchange between the IL and the aqueous buffer. They have also shown that the membrane transporter valinomycin can be incorporated and retains its functionality and cation selectivity (Fig. 29). In their other study, by means of neutron spin-echo spectroscopy and SANS, they looked at the elasticity of egg lecithin lipid vesicles in pure (protic) ILs (ethylammonium formate and ethanolammonium formate) and compared it to the vesicle’s elasticity in water (Miao et al. 2022). It turned out that the vesicle’s bending modulus is up to an order of magnitude lower in pure IL than in water, i.e., the lipid membrane is more flexible in pure IL; moreover, no change in bilayer thickness or nonpolar chain composition were detected. The effect was attributed to the dynamic association and exchange of the IL cation between the lipid membrane and the bulk liquid.

## Klähn

**Fig. 30:**
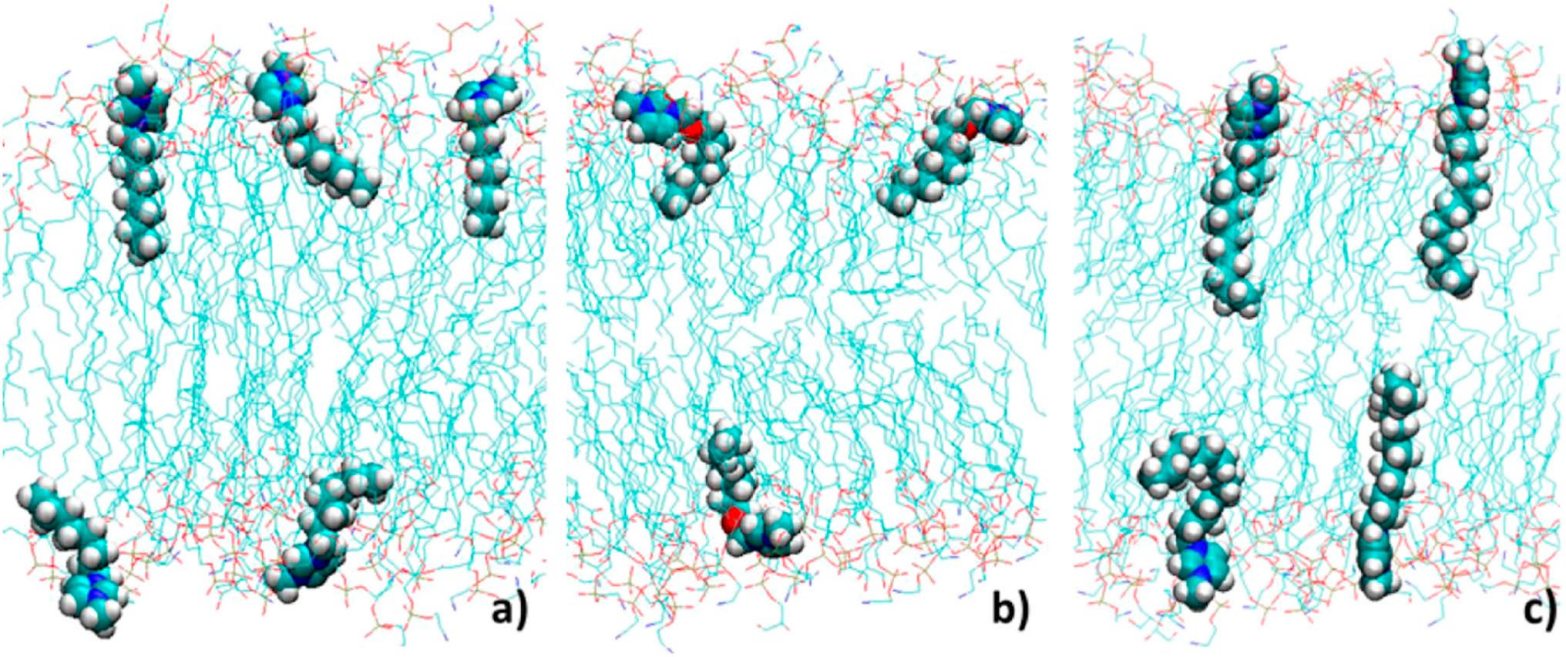
Three representative snapshots of cation insertions for **a** [omim][Cl], **b** [oxmim][Lact], **c** [tdmim][Lact]. The longer tail of [tdmim]^+^ tends to be more aligned on the average to the lipid tails. Taken from (Lim et al. 2014) and reproduced with permission from the publisher

Klähn and co-workers have contributed with two MD simulation studies to the field. In their first work (Lim et al. 2014) — to be best of my knowledge their first contribution to the field —they investigated the effect of five different imidazolium-based ILs on a lipid bilayer mimicking a bacterial plasma membrane (i.e., POPE:POPG:POPA at 80:15:5 molar ratio). They showed that IL-cations spontaneously insert into the membrane and then align their tails to the lipids’ tails, with the imidazolium-ring mostly exposed to the solvent (Fig. 30). The presence of the IL-cation in the lipid phase reduces the membrane thickness, decreases the lipid order, and generates voids filled then by curling lipids. They have also shown that not all the ILs behaves in this way, e.g., the [tdmim]^+^ increases the lipid order. In a follow-up work (Lim et al. 2015), Klähn and co-workers looked more in detail at the effect of the absorption of [omim]^+^ into the lipid-mix bacteria plasma-membrane model investigated earlier. They have shown that the IL-cation concentration inside the lipid region is about 47 times larger than in the solvent, and the absorbed cations exhibit a weak effective attraction inside the membrane at a distance of 1.3 nm, which favors the formation of cation-lipid-cation complexes. The shorter IL-chain, compared to the length of the lipid hydrocarbon chain, induces the formation of voids that destabilize the bilayer and cause its shrinkage.

## Others

The field is rich of much more studies that I try to briefly list below, following a chronological order, to give to the reader a much complete as possible overview of the field (hoping to not have missed and/or wrongly attributed any contributors, and excusing if/where this happened).

- In 2009, by means of MD simulations, Ballone and co-workers looked at the effect of [C_4_mim][Cl] and [C_4_mim][Tf_2_N] on a neutral lipid bilayer made of cholesterol (Cromie et al. 2009). The results show that both ILs get adsorbed at the water-cholesterol interface, which is more pronounced for [C_4_mim][Tf_2_N].
- In 2012, by means of MD simulations, Bingham and Ballone looked at the effect of [C_4_mim][Cl], [C_4_mim][PF_6_] and [C_4_mim][Tf_2_N] on a zwitterionic lipid bilayer made of POPC (Bingham and Ballone 2012). They observed a clear tendency for the [C_4_mim]^+^ cation to diffuse tail-first into the bilayer, and recorded the different behaviours of the anions: [Cl] ^−^ stays in water, [PF_6_] ^−^ forms a tiny layer on the surface of the bilayer, and [Tf_2_N] ^−^ precipitates out of the solution.
- In 2012, by employing a number of different approaches (e.g., DSC, confocal fluorescence microscopy, leakage assays), Jelinek and co-workers studied the interaction between several ILs with different lipid membranes, highlighting the key role played by the IL chain length and the IL headgroup (Gal et al. 2012).
- In 2012, by means of DSC and DLS, Jeon and co-workers investigated the effect of imidazolium-based ILs, of different chain length, on DMPC liposomes, showing the ability of the ILs to decrease the main phase transition temperature of the lipid and increase the liposomes’ size, with the strength of the effect positively correlating with the IL chain length (Jeong et al. 2012).
- In 2013, by means of DSC, Elliott and co-workers looked at the effect of a series of choline phosphate ILs (10 mM, pH 7.60) on the thermotropic behavior of DPPC lipid unilamellar vesicles, showing that some ILs induce minimal changes in the gel-to-liquid phase transition behaviour, while some other ILs cause the reduction of the lipid main transition temperature pointing/due to the absorption of the ILs in the lipid region (Weaver et al. 2013).
- In 2013, by means of DLS and fluorescence resonance energy transfer, Kuwabata and co-workers looked at the effect of two ILs (i.e., [emim][Lac] and [Ch][Lac]) on DOPC:SM (3:1 molar ratio) lipid vesicles, showing the ability of these ILs to induce vesicle fusion at moderate concentrations (Hayakawa et al. 2013).
- In 2015, by means of pyrene steady-state fluorescence, Galletti et al. looked at the effect of different ILs on the aggregation properties/fusion of egg-PC liposomes, showing that pyridinium-based ILs induces more liposomes’ fusion than pyrrolidinium- and imidazolium-based ILs (having the same side chain), and that anions play a marginal role (Galletti et al. 2015).
- In 2015, by means of MD simulations, Lee looked at the interaction of ILs with lipid bilayers of saturated and unsaturated chains, i.e., DMPC, POPC, and DOPC (Lee 2015). He showed that the IL-cations diffuse into the lipid region for all the lipid types under investigations, but in amounts that depend on the lipid type, e.g., about 5% and 10% in volume fraction for POPC and DMPC bilayers, respectively.
- In 2016, by means of a number of different approaches (e.g., fluorescence imaging, light and X-ray techniques, AFM), Zhu and co-workers looked at the interaction of amphiphilic ILs (around their CMC concentration) with a α-PC lipid bilayer (small unilamellar vesicles, multilamellar vesicles, and single supported bilayers), showing that the ILs could disrupt the bilayer by IL-insertion, end-capping the hydrophobic edge of the bilayer, and eventually disintegrating the bilayer at higher IL concentrations (Jing et al. 2016).
- In 2016, by means of dissipative QCM and DLS, Losada-Péres and co-workers investigated the effect of aqueous imidazolium-based IL mixtures on DMPC lipid bilayer, showing that the longer-chain ILs distort and rupture the lipid vesicles inducing the formation of supported lipid bilayers (Losada-Péres et al. 2016).
- In 2016, by means of MD simulations, Koli and Azizi studied the transport behavior of few choline-based ILs in DPPC lipid bilayer, showing that some ILs diffuse into the bilayer and decrease its thickness (Koli and Azizi 2016).
- In 2018, by means for several experimental approaches as well as MD simulations, Barros-Timmons and co-workers looked at the interaction of various imidazolium-based ILs with monolayers of DPPC:cholesterol and DPPG:cholesterol, showing significant effects for ILs containing more than six carbon atom in their alkyl chain (Mendonça et al. 2018). The effect on the bilayer containing the negatively charged DPPG lipid was stronger.
- In 2018, by means of Langmuir–Blodgett trough technique, Gonçalves da Silva looked at the effect of two phosphonium-based ILs on different lipid monolayers (e.g., DPPC and DPPG), showing that the degree of interaction depends on the charge distribution in the lipid polar group (Gonçalves da Silva 2018). Phase separation and partial miscibility have been observed, as well as the formation of IL-lipid complexes (also studied at different pH values).
- In 2020, by means of Langmuir–Blodgett apparatus and AFM, Eftaiha and co-workers studied the effect of a set of [C_n_mim][Cl] on monolayers of palmitic acid, showing that [C_16_mim][Cl] mixes non-ideally with the monolayer, stabilizes the binary mixed films, and forms nanometric propeller-like domains (Eftaiha et al. 2020). Overall, the monolayers’ compressional moduli values decrease upon increasing the IL concentration.
- In 2020, by means of MD simulations, Sun and co-workers showed the ability of amino cation-based ILs to insert into POPC lipid bilayers but not to reorient themselves as was observed instead for imidazolium-based ILs (Zheng et al. 2020). Several structural and dynamical properties of the bilayer have been computed, such as electrostatic potential, local ordering, area per lipid, volume per lipid, bilayer thickness, and lateral lipid diffusion, which all resulted significantly influenced by the IL-cations insertion.
- In 2021, by means of CPU-accelerated MD simulations, Liu et al. explored the inserting dynamics of ILs into a lipid bilayer. They found that the inserting rate increases first and then decreases nonmonotonic as side chain of the IL cation increases, peaking at 10 carbon atoms in the chain (Liu et al. 2021). On the contrary, the inserting free energy decreases with increasing the IL chain length. They explained the observed trends due to the formation of IL clusters occurring in solution for ILs with more than 10 carbons in their chain. When IL clusters are forming, the inserting rate of the IL into the lipid phase depends on the competitive balance between the cluster dissociation energy and the inserting energy.
- In 2022, by a number of different techniques including DLS and Langmuir-surface balance apparatus, Panda and co-workers looked at the effect of [bmim][Cl] on monolayers and bilayers of SPC and HSPC with cholesterol (Mandal et al. 2022). They found, for example, that the addition of the IL to the monolayer induce a decrease of the collapse pressure and an increase of the minimum molecular area. Looking at vesicles, they found that the addition of the IL increases the size of the lipid vesicles and their rigidity, and lead to the disruption of the vesicles.
- In 2022, by means of a Langmuir–Blodgett trough apparatus, Sakai and co-workers studied interaction between a hydrophobic ammonium-based IL with a monolayers of DPPC in the absence and presence of cholesterol (Kaneko et al. 2022). They have shown that the addition of the IL reduces the lipid packing and decrease the elastic modulus of the monolayer, resulting in increasing flexibility, which was more pronounced in the monolayer containing the cholesterol.

## Summary and outlook for the future

Overall, I can certainly and happily say that in the last 6 years, since my previous as well as first review of this topic (Benedetto 2017), the field has expanded both in number of researchers and research groups working in it, and in scope. A variety of different techniques, approaches, know-how, points of view, questions, backgrounds, and disciplines are all merging giving origin to a nice cross-disciplinary community of “ILs and lipid bilayers” (aka model biomembranes). Since its infancy, a lot has been done already and/but a lot still needs to be understood and investigated. For example, whereas the focus was initially on 1-lipid or 2-lipids systems, in the last few years several studies have been carried out on more complex lipid mixtures and cell-extracted lipid mixtures, which provided very interesting an important results. However, in my opinion, the biophysics and chemical-physics of 1-lipid bilayers interacting with ILs is far to be completed or (fully) understood. More detailed studies need to be carried out, screening families of ILs against family of lipids and their mixtures, which might provide useful as well as unexpected novel results, leading also to the tailoring of novel ILs. These results can inform, along with the basic research, the development of breakthrough applications in bio-nanomedicine, drug delivery, pharmacology, material science, and nano-technology. Lipid nanoparticles, for example, are one of the key actors of this century nano-bio revolution in theranostics, and ILs could certainly contribute to this endeavorer due to their strong affinity with lipids and their ability to change/control lipid bilayer properties. Last but not the least, in this (in my opinion) already fascinating and vast playground, new families of ILs are keep joining the picture and so the “game” as, for example, the families of magnetic ILs and di-cations ILs. These ILs can provide additional and unique handles to further tuning the IL-lipid bilayer interaction, broadly expanding the “rules of the game”…

## Data Availability

No datasets were generated or analysed during the current study.

## Biography: Antonio Benedetto

I am an experimental physicist and biophysicist. In 2011, I received my PhD in Physics from Messina University (Italy) defending a thesis on protein dynamics carried out at the Institute Laue-Langevin (France). I was then in Sydney (as Endeavour fellow in the Australian Centre for Neutron Scattering, formally the Bragg Institute), Dublin (as Marie-Curie fellow in UCD School of Physics), Zürich (as Marie-Curie fellow in the Paul Scherrer Institute), Dublin (as SIRG fellow in UCD School of Physics), and Rome (as Rita-Levi Montalcini fellow in URoma3 Science Department). Since 2021, I am Associate Professor in Experimental Physics & Biophysics  and lead the NanoBioPhysics Laboratory (UCD & URoma3). My main focus is on the interaction of ionic liquids with lipid bilayers and live cells, which I investigate by means of neutron scattering, atomic force microscopy, computer simulations and a number of other approaches. In 2023, I was awarded the “Michèle Auger Award” and invited to write this review.

Myself with the “2023 Michèle Auger Award” plaque.

## Biography: Michèle Auger (1963 – 2018)

Born in GrandMère, Quebec and raised in TroisRivières, Michèle Auger enrolled first in biophysics at the Université du Québec à Trois-Rivières, only to later transfer to chemistry. After obtaining her B. Sc. in 1985, Michèle joined the group of Prof. Ian C.P. Smith at the University of Ottawa/National Research Council of Canada to pursue her Ph. D. studies in biophysics. After graduating from the University of Ottawa in 1990, Michèle refined her skills in solid state NMR as a postdoctoral fellow in the group of Prof. Robert G. Griffin at the Massachusetts Institute of Technology. Michèle joined the Department of Chemistry at the University of Laval in 1991 as an Assistant Professor and recipient of an NSERC Women’s Faculty Award. She was promoted to Associate Professor in 1996 and then Professor in 2000 where she remained until 2018. Michèle’s research involved using solid state NMR to study (i) the interaction of proteins, peptides and drugs with phospholipid membranes, and (ii) biopolymers such as spider silk. Michèle served internationally on the Council of the International Union of Pure and Applied Biophysics from 2011 – 2017 and was an Editorial Board Member of Biophysical Reviews journal from 2011-2018. Brilliant, creative, dedicated to the scientific and academic communities, Michèle displayed admirable professional, ethical and leadership qualities. She is remembered as a dedicated, generous, and inspirational scientist who touched the lives of many through her friendship, teaching and kindness.

Professor Michèle Auger a much admired Editorial Board Member of Biophysical Reviews (2011 – 2018)This short foreword is adapted from a longer memorial published on the IUPAB Newsletter (IUPAB, 2019).
